# Characterization of the first beta-class carbonic anhydrase from an arthropod (*Drosophila melanogaster*) and phylogenetic analysis of beta-class carbonic anhydrases in invertebrates

**DOI:** 10.1186/1471-2091-11-28

**Published:** 2010-07-26

**Authors:** Leo Syrjänen, Martti Tolvanen, Mika Hilvo, Ayodeji Olatubosun, Alessio Innocenti, Andrea Scozzafava, Jenni Leppiniemi, Barbara Niederhauser, Vesa P Hytönen, Thomas A Gorr, Seppo Parkkila, Claudiu T Supuran

**Affiliations:** 1 Institute of Medical Technology, University of Tampere and Tampere University Hospital, Tampere, Finland; 2 School of Medicine, University of Tampere and Tampere University Hospital, Tampere, Finland; 3 Centre for Laboratory Medicine, Tampere University Hospital, Tampere, Finland; 4 Laboratorio di Chimica Bioinorganica, Università degli Studi di Firenze, Sesto Fiorentino (Firenze), Italy; 5 Vetsuisse Faculty and Zurich Center for Integrative Human Physiology (ZIHP), Institute of Veterinary Physiology, University of Zurich, Zurich, Switzerland

## Abstract

**Background:**

The β-carbonic anhydrase (CA, EC 4.2.1.1) enzymes have been reported in a variety of organisms, but their existence in animals has been unclear. The purpose of the present study was to perform extensive sequence analysis to show that the β-CAs are present in invertebrates and to clone and characterize a member of this enzyme family from a representative model organism of the animal kingdom, e.g., *Drosophila melanogaster*.

**Results:**

The novel β-CA gene, here named *DmBCA*, was identified from FlyBase, and its orthologs were searched and reconstructed from sequence databases, confirming the presence of β-CA sequences in 55 metazoan species. The corresponding recombinant enzyme was produced in Sf9 insect cells, purified, kinetically characterized, and its inhibition was investigated with a series of simple, inorganic anions. Holoenzyme molecular mass was defined by dynamic light scattering analysis and gel filtration, and the results suggested that the holoenzyme is a dimer. Double immunostaining confirmed predictions based on sequence analysis and localized DmBCA protein to mitochondria. The enzyme showed high CO_2 _hydratase activity, with a k_cat _of 9.5 × 10^5 ^s^-1 ^and a k_cat_/K_M _of 1.1 × 10^8 ^M^-^^1^s^-^^1^. DmBCA was appreciably inhibited by the clinically-used sulfonamide acetazolamide, with an inhibition constant of 49 nM. It was moderately inhibited by halides, pseudohalides, hydrogen sulfide, bisulfite and sulfate (K_I _values of 0.67 - 1.36 mM) and more potently by sulfamide (K_I _of 0.15 mM). Bicarbonate, nitrate, nitrite and phenylarsonic/boronic acids were much weaker inhibitors (K_I_s of 26.9 - 43.7 mM).

**Conclusions:**

The Drosophila β-CA represents a highly active mitochondrial enzyme that is a potential model enzyme for anti-parasitic drug development.

## Background

Carbonic anhydrases (CAs, EC 4.2.1.1) catalyze the reversible hydration of carbon dioxide according to the following reaction: CO_2 _+ H_2_O ↔ HCO_3_^- ^+ H^+ ^[1]. CAs are zinc-containing metalloenzymes, except for the ζ form, which uses cadmium [2,3] as alternative metal cofactor. Additionally γ-CAs contain iron *in vivo*, at least in anaerobic *Archaea *[4,5]. The reaction catalyzed by CAs is crucial in the regulation of acid-base balance in organisms. In addition, CAs participate in many other physiological processes such as bone resorption in vertebrates, gluconeogenesis, production of body fluids, and transport of CO_2 _and HCO_3_^- ^to name but a few [1].

To date, five different classes of CAs have been identified: α, β, γ, δ and ζ [6]. A previously known ε-CA class [7] has been reclassified as a new type of β-CA based on its crystallographic structure [8], which shows a fold nearly identical to those of the archaeal cab-type [9] and plant-type [10] β-CAs. In ζ-CA, the geometry of the active site is nearly identical to that of β-CAs, and there is also some similarity in the fold, which has led to the suggestion that even ζ-CAs might represent a distantly diverged subtype of β-CAs [3].

The ζ-CAs are found only in diatoms, and the δ-CAs in diatoms and other marine phytoplankton, whereas the three major classes (α, β and γ) are widely distributed. γ-CA orthologs are present in *Archaea, Bacteria *and plants [6,11] but have been secondarily lost in animals and fungi [12] (M. Tolvanen, unpublished observation). The α class is missing from *Archaea *(M. Tolvanen, unpublished observation) but is nearly ubiquitously present in *Bacteria *and *Eukarya*, with the exception of *Fungi*, in which only filamentous ascomycetes have α-CAs [12]. In plants and animals, α-CAs exist as numerous isoforms. For example, 16 and 15 α-CAs have been described in non-primate mammals and primates including humans, respectively [1,13]. β-CAs appear to be the class with the widest distribution as they have been described in multiple lineages throughout the tree of life, including most species belonging to the *Archaea *and *Bacteria *domains and probably all species of plants and fungi among *Eukarya *([14] and M. Tolvanen, unpublished data). β-CAs have also been characterized in a number of human pathogens such as fungi/yeasts (e.g., *Candida albicans*, *Candida glabrata, Cryptococcus neoformans *and *Saccharomyces cerevisiae*) [15-20] and bacteria (*Helicobacter pylori*, *Mycobacterium tuberculosis*, *Haemophilus influenzae*, *Brucella suis *etc.) [21-25], and their inhibition profiles by various agents such as sulfonamides, anions, carboxylates and boronic acids have been explored [15-24,26-30]. Given that β-CA genes appear to be missing in invertebrates, novel antimicrobial compounds based on the inhibition of β-CAs from pathogenic organisms may soon become available.

Although β-CAs catalyze the same reaction as α-CAs and other CA forms, important structural differences between these classes exist. First, instead of functioning as obligate monomers like most α-forms or trimers like γ-forms, β-CAs are found in many oligomerization states. Crystal structures of dimeric, tetrameric and octameric β-CAs have been reported [9,10,31]. In the active site of β-CAs, the zinc atom is coordinated by one histidine and two cysteine residues instead of three histidine residues present in α-, γ- and δ-CAs [32]. Despite this difference, β-class CAs basically share the same molecular mechanism for reversible hydration of carbon dioxide as α-class CAs [9]. β-CAs possess a highly conserved dyad comprising an aspartate and an arginine residue that seem to be crucial for the catalytic mechanism since mutation of these residues severely reduces the catalytic activity of the enzyme [33]. The aspartate makes a hydrogen bond with the Zn(II) coordinated water molecule, activating it for nucleophilic attack of the CO_2 _molecule [29].

β-CAs have been reported in many photosynthetic organisms, including plants and algae [6]. In plants, β-class CAs are strongly expressed in both roots and green tissues and are located in chloroplasts, cytoplasm and mitochondria with isozyme-specific patterns [13,34]. The cytoplasmic and chloroplastic CAs are suggested to be crucial in CO_2 _accumulation and carbon fixation. The green algae *Chlamydomonas reinhardtii *also contains β-class CAs that are localized to mitochondria [35]. In fungi like *Cryptococcus neoformans *and *Candida albicans*, β-CAs have an important role in CO_2 _sensing and, consequently, in the pathogenesis of these species [36]. In addition, many fungal β-CAs have been shown to be mitochondrial [12]. These and many other similar findings confirm that β-CAs are physiologically important enzymes with variable localization and function like α-CAs in vertebrates.

The presence of β-CAs in the animal kingdom has been controversial or ignored due to the paucity and poor quality of the available sequences [6,14]. Here we show, however, that β-CAs are widespread among invertebrates. The aim of this study was to express, purify and characterize a β-CA enzyme from fruit fly (*D. melanogaster*), a commonly used model organism in biological sciences. The recombinant enzyme was produced in Sf9 insect cells using the baculovirus/insect cell expression system. Along with the characterization of the first arthropod β-CA, this study also describes its inhibition profile with inorganic anions. These results might open new strategies for developing novel anti-parasitic drugs against common diseases like schistosomiasis and malaria.

## Results

### Sequence analysis

We found β-CAs in all complete non-chordate animal genomes and in almost all invertebrates with at least 20,000 EST sequences in the NCBI database, plus in some with fewer sequences. We confirmed the existence of β-CA sequences in *Placozoa, Cnidaria, Platyhelminthes, Nematoda, Arthropoda*, and *Annelida*, and even in many classes of *Deuterostomia*, namely in *Hemichordata, Echinodermata*, and *Xenoturbellida*. The only major taxon of *Protostomia *with poor evidence for β-CA is *Mollusca*, in which we found only one EST to match 55 residues in other β-CAs. Of special interest were numerous pathogenic helminth species with complete β-CA sequences or substantial fragments, namely, filaria-causing *Brugia malayi*, mouse whipworm *Trichuris muris*, dog hookworm *Ancylostoma caninum*, and the flukes *Schistosoma mansoni *and *Schistosoma sinensis*.

In the case of chordates, the existence of a functional β-CA is currently unclear. The genomes and NCBI EST sequence collections (as of 1^st ^Oct, 2009) of *Ciona intestinalis *and *Ciona savignyi *(tunicates) lack β-CA, whereas we found two recognizable but incomplete β-CA sequences in the genome of the cephalochordate *Branchiostoma floridae *(of one locus from both haplotypes in the genome). The encoded proteins seem to lack more than 60 residues at the N-terminus, including the active site. In addition, there are two even less complete partial EST transcripts, GenBank BW824885 and BW803919. The latter contains an unrelated sequence in place of the active-site-containing exon. We can only conclude that it remains an open question whether β-CA in *B. floridae *is a pseudogene or an incompletely sequenced active gene.

**Figure 1:**
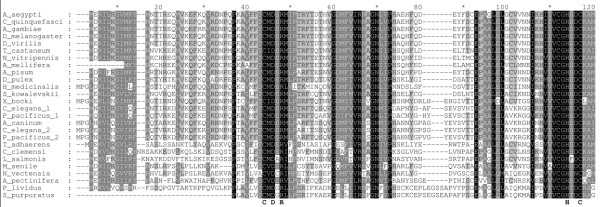
**Alignment of 26 β-CA sequences from invertebrate species**. Only the first 120 residues are shown. Active-site residues C, D, R, H and C are highlighted below the alignment. These residues are found in all β-CAs. See Table 1 for identification of species.

**Figure 2:**
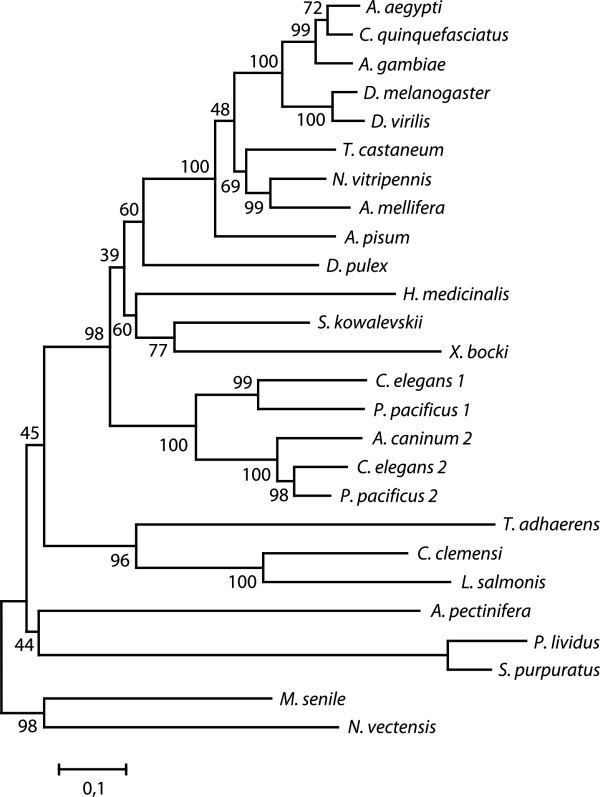
**Minimum-evolution tree of 26 invertebrate β-CA sequences**. Bootstrap consensus tree of 1,000 replicates. The percentage of replicate trees in which the associated taxa clustered together in the bootstrap test are shown next to the branches. See Table 1 for identification of species.

Our survey discovered and assembled 38 seemingly complete and correct β-CA sequences from the genome and sequence databases of 33 metazoan species, including improved gene models for sequences already in sequence databases. Fragmentary β-CA sequences were found in additional 22 species. Multiple sequence alignment of all animal β-CAs shows perfect conservation of the known active site motifs CxDxR and HxxC and several other key residues. Figure 1 shows an alignment of the first 120 residues of selected β-CA sequences, including the N-terminal mitochondrial targeting peptide and active site regions (See Table 1 for identification of species). Of the active-site residues indicated below the alignment, two cysteines and one histidine are zinc-binding residues. Additional files 1 and 2 show the full alignment of the same sequences and of all of the identified sequences, respectively. The phylogenetic tree of the selected animal β-CA sequences is shown in Figure 2 (See Table 1 for identification of species). The tree indicates that the duplication of β-CA genes in nematodes is specific for the nematode lineage. Out of the two copies, the one labeled BCA2 is more strongly conserved, as shown by shorter branches in the tree of Figure 2 and in trees we made with all available sequences. The placement of the β-CA from acorn worm (*S. kowalevskii*, a hemichordate) seems contrary to conventional invertebrate taxonomy, but since some of the bootstrap values are under 50%, the tree is not perfectly resolved outside the insect and nematode blocks.

**Table 1:** Identification of sequences and species in the sequence alignment and phylogenetic tree

Abbreviation	Full name	Common name and classification	gi or accession numbers
**A_aegypti**	*Aedes aegypti*	Yellow fever mosquito, insects	157110803 + 77891004
**C_quinquefasciatus**	*Culex quinquefasciatus*	Southern house mosquito, insects	170043321
**A_gambiae**	*Anopheles gambiae*	Malaria mosquito, insects	57968460
**D_melanogaster**	*Drosophila melanogaster*	Fruit fly, insects	24645213
**D_virilis**	*Drosophila virilis*	Fruit fly, insects	194152748
**T_castaneum**	*Tribolium castaneum*	Red flour beetle, insects	91084165
**N_vitripennis**	*Nasonia vitripennis*	Wasp, insects	156547528
**A_mellifera**	*Apis mellifera*	Honeybee, insects	110764310
**A_pisum**	*Acyrthosiphon pisum*	Pea aphid, insects	193713675
**D_pulex**	*Daphnia pulex*	Water flea, crustaceans	FE417346 + FE409868
**H_medicinalis**	*Hirudo medicinalis*	Medical leech, Annelid worms	EY481200 + EY505051 + EY490477
**S_kowalevskii**	*Saccoglossus kowalevskii*	Acorn worm, hemichordate	187043763
**X_bocki**	*Xenoturbella bocki*	Xenoturbella, Xenoturbellidae	117195962
**C_elegans_1**	*Caenorhabditis elegans *BCA1	Nematode	NP_741809.1
**P_pacificus_1**	*Pristionchus pacificus *BCA1	Nematode	FG098717 + GeneWise
**A_caninum**	*Ancylostoma caninum*	Dog hookworm, nematodes	FC551456 + FC550353
**C_elegans_2**	*Caenorhabditis elegans *BCA2	Nematode	NP_001041015
**P_pacificus_2**	*Pristionchus pacificus *BCA2	Nematode	FG106379 + GeneWise
**T_adhaerens**	*Trichoplax adhaerens*	Trichoplax, Placozoa	190581916
**C_clemensi**	*Caligus clemensi*	Sea louse, crustaceans	225719368
**L_salmonis**	*Lepeophtheirus salmonis*	Sea louse, crustaceans	225713548
**M_senile**	*Metridium senile*	Sea anemone, Cnidaria	FC835283
**N_vectensis**	*Nematostella vectensis*	Sea anemone, Cnidaria	XP_001632619
**A_pectinifera**	*Asterina pectinifera*	Starfish, Echinodermata	DB424979 + DB440523
**P_lividus**	*Paracentrotus lividus*	Sea urchin, Echinodermata	139313180 + 139245724
**S_purpuratus**	*Strongylocentrotus purpuratus*	Purple sea urchin, Echinodermata	XP_001189115

No β*-CA *sequences exist in vertebrate genomes. A false positive is found in the *X. tropicalis *genome, scaffold_1719, which we attribute to bacterial contamination, since the β-CA sequence and every other gene in this scaffold are highly similar (>80% identical) with known genes of *Pseudomonas*-related bacteria.

All of the complete sequences are classified as β-CA domains of type "BCA_CladeB" in the NCBI Conserved Domain Database. All plant β-CAs are also classified as BCA_CladeB domains, so animal β-CAs can well be said to be "plant-type β-CAs". There are also hundreds of bacterial β-CA sequences of type "BCA_CladeB", and some of them cluster closest to animal sequences, and some closest to plant sequences in phylogenetic trees (data not shown).

### Expression of β-CA in Sf9 insect cells

**Figure 3:**
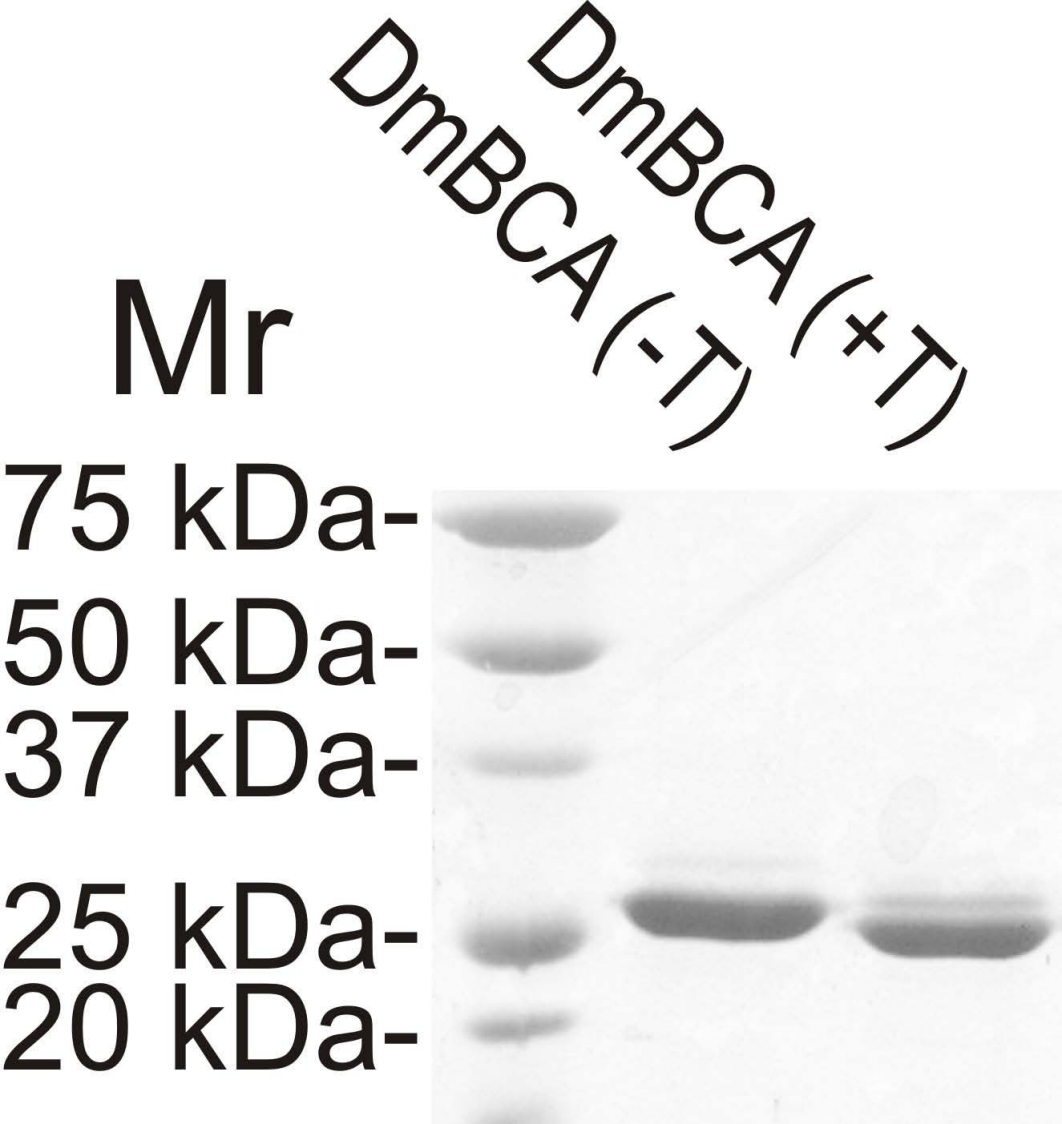
**SDS-PAGE of the produced DmBCA**. T = thrombin treatment.

Sf9 insect cells were transfected with the β-CA gene (*DmBCA*) obtained from *D. melanogaster *cDNA. The amount of protein obtained from 500 ml of culture supernatant was approximately 1 mg. According to SDS-PAGE, the relative molecular mass of DmBCA was approximately 28 and 27 kDa before and after thrombin treatment, respectively (Figure 3).

### Subcellular localization of DmBCA

When the collection of 38 complete animal β-CA sequences was analyzed for subcellular targeting with TargetP, 22 sequences were predicted to be mitochondrial, with low reliability, and most of the remaining sequences were predicted to be cytoplasmic, again with low reliability. The results from WolF PSORT, Secretome 2.0 and MitoProt II v. 1.101 also supported the hypothesis that it is a mitochondrial enzyme, with a possibility for non-classical secretion (without a signal peptide) suggested by Secretome 2.0. A mitochondrial targeting signal sequence is also predicted in the N-terminus (prediction lengths varying from 14 in MitoProt to 49 in TargetP). Based on these findings, a DmBCA-GFP construct was designed to study the subcellular localization of the protein in Sf9 cells.

**Figure 4:**
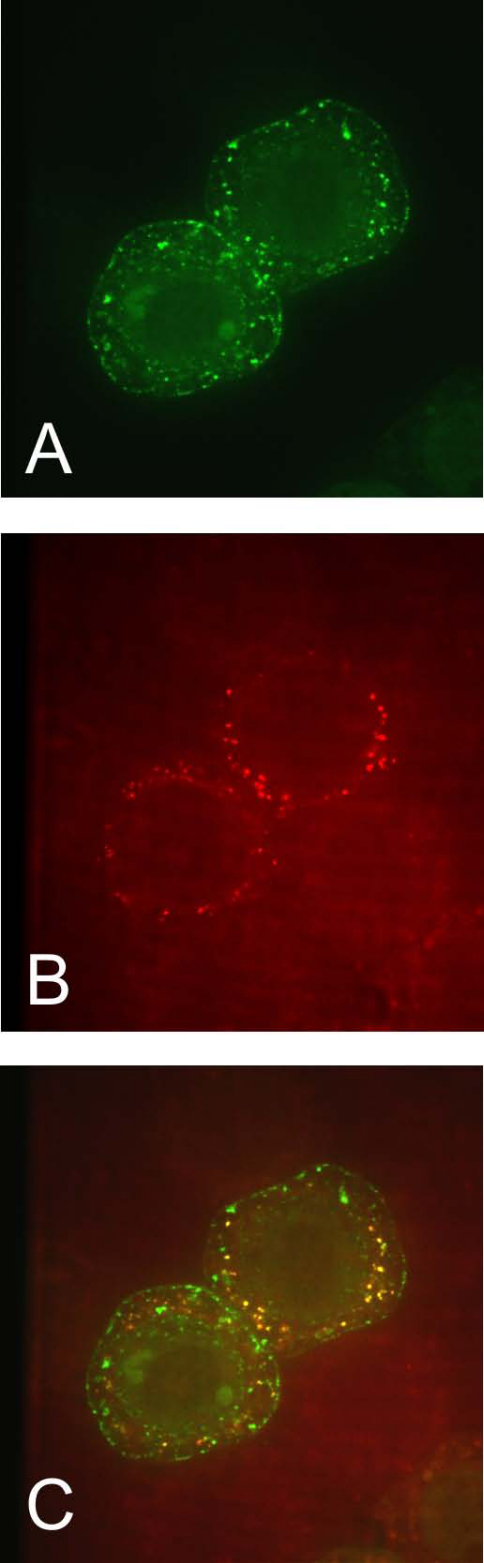
**Double immunofluorescence staining of DmBCA-GFP and mitochondria in Sf9 cells**. Green fluorescent signal shows the location of the DmBCA-GFP fusion protein (A), and the red color labels the mitochondria (B). The yellow color in panel C indicates the colocalization of these two labels.

According to our experiments on the DmBCA-GFP fusion protein, DmBCA is indeed a mitochondrial protein, supporting the predictions made by bioinformatic tools. Figure 4A shows the DmBCA-GFP recombinant protein in Sf9 insect cells in which the positive signal was located in intracellular granular structures. Figure 4B shows the same cells labeled with a mitochondrial marker, MitoTracker Red CMXros™. Figure 4C presents an overlay of the previous panels, demonstrating the colocalization of DmBCA-GFP and MitoTracker Red CMXros™.

### Catalytic activity and inhibition of DmBCA

DmBCA purified from Sf9 cells was kinetically analyzed in the presence or absence of acetazolamide or inorganic anions. The kinetic parameters of DmBCA (k_cat _and k_cat_/K_m_) were then compared with those of the thoroughly investigated CAs such as the cytosolic and ubiquitous human isozymes α-CA I (HCA I) and II (HCA II), as well as the recently described fungal β-CAs, *C. neoformans *Can2, *C. albicans *Nce103, *S. cerevisiae *CA (ScCA) and *C. glabrata *CA (CgCA). All of these fungal enzymes are orthologous to *Nce103*, a protein found in all fungi/yeasts studied to date (Table 2).

**Table 2:** Kinetic parameters for the CO2 hydration reaction catalyzed by various CAs

Isozyme	Activity level	**k**_**cat **_**(s**^**-1**^**)**	**k**_**cat**_**/K**_**m **_**(M**^**-1**^**s**^**-1**^**)**	**K**_**I **_**(nM)**^**c**^
**HCA I**^**a**^	moderate	2.0 × 10^5^	5.0 × 10^7^	250
**HCA II**^**a**^	very high	1.4 × 10^6^	1.5 × 10^8^	12
**Can2**^**a**^	moderate	3.9 × 10^5^	4.3 × 10^7^	10.5
**Nce103**^**a**^	high	8.0 × 10^5^	9.7 × 10^7^	132
**ScCA**^**a**^	high	9.4 × 10^5^	9.8 × 10^7^	82
**CgCA**^**a**^	moderate	3.8 × 10^5^	4.8 × 10^7^	11
**DmBCA**^**b**^	high	9.5 × 10^5^	1.1 × 10^8^	49

Kinetic parameters for the human cytosolic isozymes human HCA I and II (α-class CAs) at 20°C and pH 7.5 in 10 mM HEPES buffer and 20 mM Na_2_SO_4_, in addition to the β-CAs Can2, Nce103 (from *C. neoformans *and *C. albicans*, respectively), ScCA (from *S. cerevisiae*), CgCA (from *C. glabrata*) and DmBCA (from *Drosophila melanogaster*) measured at 20°C, pH 8.3 in 20 mM Tris buffer and 20 mM NaClO_4 _are shown. Inhibition data with the clinically used sulfonamide acetazolamide (5-acetamido-1,3,4-thiadiazole-2-sulfonamide) are also provided.

^a^Reference [30]

^b^This work

^c^acetazolamide

One can appreciate from the data shown in Table 2 that DmBCA, similar to the other recently investigated α- and β-CAs, possesses considerable CO_2 _hydratase activity. A k_cat _of 9.5 × 10^5 ^s^-1 ^and a k_cat_/K_m _of 1.1 × 10^8 ^M^-1^s^-1 ^make DmBCA one the most efficient β-CA described to date. Data from Table 2 also show that DmBCA was appreciably inhibited by the clinically-used sulfonamide acetazolamide (5-acetamido-1,3,4-thiadiazole-2-sulfonamide), with an inhibition constant of 49 nM. Table 3 shows the DmBCA inhibition screening data with anionic physiological species (such as chloride, bicarbonate, sulfate, etc.) as well as other non-physiological anions. Note that similar to other investigated α- and β-CAs, DmBCA has an inhibition profile with anions characteristic only for this enzyme. Several species such as bicarbonate, nitrate, nitrite, perchlorate, phenylboronic acid and phenylarsonic acid behaved as weak inhibitors (K_I_s in the range of 22.4 - 200 mM), whereas other anions inhibited the enzyme more potently (e.g., cyanide, cyanate, and sulfamide, with inhibition constants in the range of 150 - 730 μM).

**Table 3:** Inhibition constants of anionic inhibitors against various CAs

Inhibitor			**K**_**I **_**[mM]**^**#**^		
	HCA II	Nce103	ScCA	CgCA	DmBCA
**F**^**-**^	>300	0.69	2.85	0.36	0.80
**Cl**^**-**^	200	0.85	0.85	0.58	0.97
**Br**^**-**^	63	0.94	0.0108	27	1.04
**I**^**-**^	26	1.40	0.0103	42.4	1.18
**CNO**^**-**^	0.03	1.18	31.7	0.60	0.73
**SCN**^**-**^	1.6	0.65	55.6	0.73	1.28
**CN**^**-**^	0.02	0.011	16.8	1.12	0.67
**N**_**3**^**-**^_	1.5	0.52	27.9	1.03	1.12
**HCO**_**3**^**-**^_	85	0.62	0.78	0.086	26.9
**NO**_**3**^**-**^_	35	0.69	13.9	0.097	43.7
**NO**_**2**^**-**^_	63	0.53	0.46	0.088	28.6
**HS**^**-**^	0.04	0.37	0.33	0.10	1.01
**HSO**_**3**^**-**^_	89	0.54	0.33	0.10	1.29
**SO**_**4**^**2-**^_	>200	14.15	0.58	0.58	1.36
**ClO**_**4**^**-**^_	>200	>200	>200	>200	>200
**H**_**2**_**NSO**_**2**_**NH**_**2**_	1.13	0.30	0.0087	0.42	0.15
**H**_**2**_**NSO**_**3**_**H**^**§**^	0.39	0.70	0.84	0.11	2.45
**Ph-B(OH)**_**2**_	23.1	30.8	38.2	0.10	22.4
**Ph-AsO**_**3**_**H**_**2**^**§**^_	49.2	30.8	0.40	0.098	32.6

Inhibition constants against isozymes HCA II (α-CA class), and the β-CAs Nce103 (from *C. albicans*), ScCA (from *S. cerevisiae*), CgCA (from *C. glabrata*) and DmBCA (*D. melanogaster*) for the CO_2 _hydration reaction at 20°C are shown.

^§^As sodium salt.

^# ^Errors in the range of 5-10% of the shown data, from three different assays, by a CO2 hydration stopped-flow assay.

### Dynamic light scattering and gel filtration analysis

The hydrodynamic diameters of the proteins were measured by dynamic light scattering (DLS). At a temperature range from 4°C to 37°C the hydrodynamic diameter of DmBCA was 6.3 ± 0.8 nm and the diameter was found to slightly increase during the heating. According to the hydrodynamic diameter the average molecular weight of DmBCA was estimated to be 48.9 kDa, the lowest estimate being 35.4 kDa, and the highest 64.9 kDa (n = 30). When temperature was raised above 43°C the hydrodynamic diameter of DmBCA started to clearly increase and DmBCA had a transition state at temperature range 46-49°C, where large protein aggregates appeared. This might be associated with protein denaturation.

At the temperature range from 4°C to 37°C the hydrodynamic diameter of HCA II was 3.9 ± 0.6 nm leading to estimated average molecular weight of 16.1 kDa, the lowest estimate being 10.8 kDa, and the highest 22.8 kDa (n = 12). When HCA II was heated from 4°C to 43°C there was a slight increase in hydrodynamic diameter. However, no transition states were observed.

The estimated molecular weights determined by DLS are in agreement with the values obtained by analytical gel filtration which gave a molecular weight estimate for DmBCA of 38.1 ± 3.0 kDa and for HCA II of 23.4 ± 4.0 kDa. The molecular weight of HCA II monomer is 29 kDa. Therefore, gel filtration analysis appears to underestimate the molecular size of CAs. Overall, DLS and gel filtration analyses suggest dimeric state for DmBCA whereas HCA II appears predominantly monomeric in solution.

## Discussion

In the present study, we identified and characterized a novel β-CA enzyme (DmBCA) from an arthropod, *D. melanogaster*. Although β-CAs have been previously reported in *Archaea *and *Bacteria *domains, in addition to plants and fungi in *Eukarya*, our results suggest the widespread occurrence of at least a single-copy β-CA gene among animal species distinct from chordates. The loss of β*-CA *gene in the chordate lineage may have occurred either in the last common ancestor of all chordates or in the last common ancestor of tunicates and craniates. Whether cephalochordates have a functional β*-CA *gene remains an unresolved question.

Using bioinformatic tools, we discovered a single β*-CA *gene in most invertebrates with reasonable sequence coverage. The exception is nematodes, which seem to have two β*-CA *genes in their genomes. A very recent article [37] reported the cloning and characterization of *C. elegans *β-CAs and the authors found that one of the two isoforms, BCA-1, has no activity and does not work in complementation assay. This might, however, be due to incorrect sequence: the sequence they show for *C. elegans *BCA-1 contains the coding sequence of the preceding *MTP18 *gene fused to the β*-CA *reading frame. This sequence is a result of an incorrect gene prediction which has no support at the mRNA level and which remained in WormBase and UniProt until May 2008, subsequently corrected from our initiative. The previously fused UniProt entry Q8T3C8 now represents only MTP18, and a new entry BCA1_CAEEL contains the more plausible BCA-1 sequence. Since *C. elegans *BCA-1 has all of the active-site residues and is also well conserved in other nematodes, we think it is probable that both isozymes in nematodes would be functional β-CAs.

Our larger phylogenetic analysis (M. Tolvanen, unpublished data) and NCBI domain classification of animal β-CAs clearly show them to be "plant-type" β-CAs. The "plant-type" group also includes hundreds of bacterial β-CAs, and this group contains plant/bacterial and animal/bacterial subgroups. The presence of such polyphyletic subgroups may indicate horizontal gene transfer, and further investigations are underway to study this phenomenon.

Fasseas *et al. *[37] state that the 3D structure of their *C. elegans *β-CA models would resemble archaeal "cab-type" β-CAs, but we cannot agree with their conclusion. In our eyes, the models are nearly identical to the pea BCA (panel a in their Figure 2), and in our hands SwissModel http://swissmodel.expasy.org/ in fact chooses the pea β-CA structure as a template for both insect and nematode β-CAs.

In the recent paper by Fasseas *et al. *[37], the enzyme activity of *C. elegans *Y116A8C.28 was reported to be typical for β-CAs, with a k_cat _and k_cat_/K_m _of 2.77 × 10^4 ^s^-1 ^and 6,383 × 10^5^M^-1^s^-1^, respectively. These values are significantly lower than the k_cat _of 9.5 × 10^5 ^s^-1^, and k_cat_/K_m _of 1.1 × 10^8 ^M^-1^s^-1 ^that we report here for DmBCA. The enzymatic activity of DmBCA is one of the highest reported so far for a β-CA, suggesting an important physiological role for this enzyme.

Our studies with different anionic CA inhibitors did not reveal specific inhibitors of DmBCA, which is in fact normal for such simple inorganic ions [38]. The inhibition profile of DmBCA was unique for this enzyme, however, and differed significantly from other β-CAs studied previously.

Indeed, fluoride, chloride, cyanate, cyanide and sulfamide showed submillimolar inhibitory activity against DmBCA, with inhibition constants in the range of 150 μM - 970 μM. Another groups of anions, including bromide, iodide, thiocyanate, azide, hydrogen sulfide, bisulfite and sulfate showed inhibition constants close to 1 mM (K_I_s in the range of 1.01 - 1.36 mM), thus making them weak DmBCA inhibitors. Other anions such as bicarbonate, nitrate and nitrite, as well as the non-anionic species phenylboronic acid and phenylarsonic acid, were much less effective inhibitors, with inhibition constants of 22.4 - 43.7 mM. It is interesting to note that bicarbonate, a CA substrate, is a weak DmBCA inhibitor, but it appreciably inhibits the fungal enzymes Nce103 (C. Albicans), ScCA and CgCA, with K_Is _in the submillimolar range (of 86 μM - 0.78 mM). Even though the low apparent K_I _value for bicarbonate could be a reflection of allosteric inactivation of the enzyme ([39,40]), the value is still in the same range with the other anion inhibitors, suggesting that their mechanism of inhibition might be quite similar. The present inhibition data may suggest a different physiological role for DmBCA in the insect compared to the corresponding β-CAs in fungal or yeast species studied previously. One should also note that DmBCA has a completely different inhibition profile not only compared to other β-CAs but also compared to the highly investigated α-CA of human origin, HCA II (Table 3).

Animal β-CAs constitute a separate subgroup in the β-CA family according to our bioinformatic analysis. Because the main target, β-CA, is present in many parasites and disease carrying vectors but absent in humans, this discovery clearly carries the significant potential for the design of parasite-specific β-CA inhibitors. Such drugs would presumably combine high specificity with a low incidence of side-effects in humans. These drugs could, for example, provide novel opportunities to restrict malaria propagation and to treat patients suffering from helminth infections.

Predictions of subcellular localization placed most, but not all, animal β-CAs in the mitochondria. Our hypothesis is that all of them will be mitochondrial based on three main reasons. First, we have direct experimental evidence of mitochondrial localization of DmBCA. Second, all of the TargetP predictions were either mitochondrial or cytoplasmic, both with low-reliability. Third, the neural network used in TargetP is trained with human and Arabidopsis mitochondrial proteins; thus, it is perhaps not perfectly adjusted to detect the signals in invertebrate mitochondrial transit peptides. Because of this mitochondrial localization of animal β-CAs, we speculate that the β-CA gene in chordates might have been silenced and ultimately lost after the emergence of mitochondrial α-CAs (CA V) to substitute for this function. The presence of an active CA in mitochondria has been linked to maintaining fundamental metabolic functions such as gluconeogenesis, ureagenesis and lipogenesis [1,41]. Although our experiments were not focused on the role of the DmBCA enzyme, its mitochondrial localization and high enzymatic activity suggest that knockdown studies in *D. melanogaster *are warranted to further investigate the physiological function of β-CAs in animals.

The expression of DmBCA has been studied at mRNA level in two publicly available DNA microarray datasets. In FlyAtlas (http://flyatlas.org/atlas.cgi?name=CG11967-RA, [42]) expression levels in 17 adult and 8 larval *D. melanogaster *tissues are given. The highest upregulated values in adult are found in spermatheca (female), fat body, and heart. In larval tissues (third instar) downregulated or average expression levels are seen for all tissues. A time course study published in FlyBase (http://flybase.org/reports/FBgn0037646.html, under Microarray data, Personal communications to FlyBase, Gaurav et al. 2008), shows upregulated expression of DmBCA in early larval stages and late in metamorphosis, but downregulated levels in embryonal, late larval, early metamorphosis, and adult stages. The specific temporal and tissue patterns of expression suggest relevant functional and/or physiological roles for DmBCA.

## Conclusions

In conclusion, fruit fly (*Drosophila melanogaster*) β-CA (DmBCA) is an active mitochondrial enzyme for the physiological reaction catalyzed by CAs, the hydration of CO_2 _to bicarbonate and protons. It is inhibited by various inorganic anions, boronic/arsonic acids or sulfonamides. Mammals do not possess β-class CAs, but these enzymes are widespread throughout the phylogenetic tree, making them exciting new targets for parasitic drug development. Indeed, β-CAs are found in many pathogenic organisms and pathogen vectors of the animal kingdom, including the malaria mosquito *Anopheles*, the yellow fever mosquito *Aedes*, the filariasis vector *Culex, Ancylostoma *hookworms,* Brugia *filarial nematodes, the blood fluke *Schistosoma mansoni *and the liver fluke *Schistosoma sinensis*. Because animal-derived β-CAs probably have a different predicted structure compared to the β-CAs investigated so far in detail from Archaea, plants, algae and fungi, as well as the α-CAs, specific inhibitors against these enzymes could be designed with minimal effect on human CAs and normal bacterial flora.

## Methods

### Bioinformatic analysis

Taking advantage of the known pea β-CA (UniProt P17067) as an initial query, and subsequently the found invertebrate β-CAs (*D. melanogaster*, AAF54311; *C. elegans *CAJ43916), the animal CA sequences were retrieved from NCBI protein databases using Blast [43]http://blast.ncbi.nlm.nih.gov/Blast.cgi and from complete genomes at the UCSC Genome Bioinformatics Site http://genome.ucsc.edu using BLAT search algorithms [44]. Hits were taken through iterated cycles of multiple sequence alignment (ClustalW; [45]), evaluation and revision. For revision, sequences with poorly matching or missing regions were subjected to gene model generation with GeneWise ([46]; http://www.ebi.ac.uk/Tools/Wise2/), taking the genomic sequences from the UCSC site. EST and mRNA sequence data from NCBI were used to confirm gene models, sometimes to bridge gaps or fill ends in the genomic sequences, and to discover and assemble β-CAs from less than genome-wide sequenced organisms.

Phylogenetic trees were constructed from the multiple sequence alignments using MEGA 4 [47]. Preliminary Neighbour-Joining trees [48] were constructed with all sequences, and a representative set of 26 sequences was then selected for a final tree, eliminating excessive insect and nematode sequences and severely incomplete sequences. The final tree was inferred using the Minimum evolution method [49] from a multiple sequence alignment containing columns corresponding to positions 31 to 255 (of 255) of DmBCA. The bootstrap consensus tree inferred from 1,000 replicates [50] was taken to represent the evolutionary history of the analyzed sequences. The tree was drawn to scale, with branch lengths proportional to the evolutionary distances used to infer the phylogenetic tree and rooted using the Cnidarian sequences as outgroup. The evolutionary distances were computed using the Poisson correction method and are presented in the units of the number of amino acid substitutions per site. The minimum evolution tree was searched using the Close-Neighbor-Interchange algorithm [51] at a search level of 3. The Neighbor-Joining algorithm [48] was used to generate the initial tree. All positions containing alignment gaps and missing data were eliminated only in pairwise sequence comparisons (Pairwise deletion option).

Predictions of subcellular localization were made using TargetP v1.1 ([52]; http://www.cbs.dtu.dk/services/TargetP/), Secretome 2.0 http://www.cbs.dtu.dk/services/SecretomeP/, WolF PSORT, http://wolfpsort.org/ and MitoProt II v. 1.101 http://ihg2.helmholtz-muenchen.de/ihg/mitoprot.html. Conserved domain types were identified at the NCBI Conserved Domains Database http://www.ncbi.nlm.nih.gov/Structure/cdd/cdd.shtml.

### Construction of recombinant baculoviruses

Two constructs were engineered to study functional aspects of *D. melanogaster *β-CA (DmBCA). The first one contained a *GFP *(Green Fluorescent Protein) sequence fused C-terminally to the *DmBCA *cDNA for detection of the recombinant protein with confocal microscopy. The second construct contained a C-terminal histidine tag for protein purification. Both constructs contained full length β-CA gene, including the proposed N-terminal signal sequence. *Bgl*II and *Xho*I restriction sites and a thrombin cleavage site (for tag removal) were introduced into both constructs.

Total RNA extracted from *D. melanogaster *S2 cells (TRIzol^® ^reagent and protocol, Invitrogen) was precipitated using sodium acetate at a final concentration of 100 mM and 100% ethanol. The solution was centrifuged at 13,000×*g *for 15 min at +4°C. The RNA sample was washed once with 70% ethanol and recentrifuged in the same manner. The sample was evaporated at room temperature and then suspended in sterile water. Precipitated RNA was transcribed into cDNA using a First strand cDNA Synthesis Kit #K1612 (Fermentas) according to the manufacturer's instructions.

The *DmBCA *gene was identified and amplified from cDNA by PCR using Phusion™ Hot Start High Fidelity DNA Polymerase (Finnzymes, Espoo, Finland). Sequence-specific primers were ordered from Sigma-Aldrich (St. Louis, MO). The forward primer was 5'-ATGGAGCGTATTTTGAGGGGAATC-3' (F1), and the reverse primer was 3'-CTACGAATAGAATCTTCTGACCTC-5' (R1). PCR was performed in a PTC 2000 thermal cycler (MJ Research, Waltham, MA), and the program consisted of a single 98°C denaturation step for 30 s, followed by 33 cycles of denaturation at 98°C for 10 s, annealing at 53°C for 15 s and extension at 72°C for 25 s. A final extension was performed at 72°C for 5 min. The PCR product band was separated from the gel and dissolved using Illustra™ GFX PCR DNA and GEL Band Purification Kit (GE Healthcare Life Sciences, Buckinghamshire, UK).

To engineer the *DmBCA-GFP *construct, the sequences of *DmBCA *and *GFP *were first amplified separately using sequence specific primers. The templates used for *DmBCA *and *GFP *were *DmBCA *gene product obtained from cDNA and pEGFP-N1™ Vector (Clontech), respectively. The constructs were then combined using PCR reactions whose R2 and F3 primer sequences partly overlapped (bolded in the primer sequences, see below). This allowed the ends of the PCR products to recognize each other. The forward primer used for *DmBCA *amplification was 5'- GGCCAGATCTATGGAGCGTATTTTGAGGGGA-3' (F2), and the reverse primer was 5'-**CACGGAACCACGGGGCACCAG**CGAATAGAATCTTCTGACCTC-3' (R2). The bolded sequence was designed to recognize the thrombin site and part of the *GFP *PCR product, while the other half recognized the *DmBCA *PCR product.

The forward primer used for GFP amplification was 5'-**TCGCTGGTGCCCCGTGGTTCCGTG**AGCAAGGGCGAGGAGCTG-3' (F3), and the reverse primer was 5'-CCGCTCGAGTTACTTGTACAGCTCGTCCAT-3' (R3). The bolded sequence in the F3 primer was designed to recognize the thrombin site and part of the *DmBCA *PCR product, while the other half recognized the *GFP *PCR product. The PCR program was as follows: 98°C for 2 min; then 33 cycles of 98°C for 10 s, 55°C for 15 s, and 72°C for 30 s; and finally 72°C for 7 min.

Both PCR products were run on agarose gel, purified and used as templates in the next PCR reaction. The PCR program was as follows: 98°C for 2 min; then 33 cycles of 98°C for 10 s, 55°C for 15 s and 72°C for 40 s; and finally 72°C for 8 min. The forward primer used in this PCR was (F2), and the reverse primer was (R3). The extra sequence overlaps at the 3' end of *DmBCA *and at the 5' end of the *GFP *allowed these PCR products to anneal to each other.

The PCR product was run on an agarose gel, and the obtained band was purified. pFastBac™ 1 plasmid (Invitrogen) and the PCR product were digested at +37°C overnight with *BamHI *and *XhoI *restriction enzymes (New England Biolabs). The digested plasmid and *DmBCA-GFP *construct were purified and then ligated overnight at +4°C using T4 DNA ligase (New England Biolabs). The ligated product was transformed into TOP10 bacteria (Invitrogen). Overnight cultures (8 ml) were made from these colonies, and plasmids were purified using a QIAprep Spin Miniprep Kit™ (Qiagen, Hilden, Germany). Sequencing was performed to verify the validity of the *DmBCA-GFP *construct. The construction of baculoviral genomes encoding the recombinant proteins has been described previously [53].

For recombinant protein production, the *DmBCA *gene construct with a C-terminal polyhistidine tag of six histidines was constructed and cloned into the pFastBac1™ vector. The forward primer used in the initial amplification of the *DmBCA *gene was (F2), and the reverse primer was 5'- GCCCTCGAGTTA**ATGGTGGTGATGGTGGTG**GGAACCACGGGGCACCAGCGAATAGAATCTTCTGACCTC -3' (R4). The latter primer contains nucleotide repeats to create the polyhistidine tag (bolded in the primer sequence). The PCR program was as follows: 98°C for 60 s; then 35 cycles of 98°C for 10 s, 66°C for 15 s, and 72°C for 60 s; and finally 72°C for 5 min. Otherwise, the construct was made essentially in the same way as the *DmBCA-GFP *construct described above.

### Study of the subcellular localization of DmBCA

A total of 100 μl of Sf9 insect cells (2 million cells/ml) were infected with 10 μl of baculovirus stock. The cells were kept at +27°C in incubator for three days in Lab-Tek™ Chamber Slide™ System™ plates (Nunc). The medium was then removed, and the cells were incubated in 600 μl of medium containing 100 nM Mitotracker Red CMXros™ (Invitrogen) for 20 minutes at +27°C. The cells were washed three times with 600 μl of medium and kept at +27°C in an incubator for two hours. The cells were then washed with PBS, fixed with 4% paraformaldehyde for five minutes and washed again with PBS. The cells were mounted in VectaShield^® ^Mounting Medium (Vector Laboratories), covered with cover slips and analyzed using a confocal scanning laser microscope (Perkin Elmer-Cetus/Wallac UltraView LCI system™) with two different wavelengths: 488 nm for GFP detection and 579 nm for MitoTracker™. Image acquisition was performed with an Andor iXon™ DV885 EMCCD camera and the Andor iQ™ software (Andor).

### Production and Purification of Recombinant Insect β-CA

The Sf9 insect cells were grown in HyQ SFX-Insect serum-free cell culture medium (HyClone, Logan, UT) in an orbital shaker at 27°C (125 rpm) for three days after infection. Although much of the DmBCA was associated with the cell pellet, protein purification was performed after centrifugation (5000 × g, 20°C, 8 min) from the supernatant and yielded highly pure DmBCA protein for characterization. Purification was performed using the Probond™ Purification System (Invitrogen) under native binding conditions, with wash and elution buffers made according to the manufacturer's instructions. The purification procedure per 500 ml of insect cell medium was as follows: 1 liter of native binding buffer and 25 ml of the nickel-chelating resin were added to the medium, and the His-tagged protein was then allowed to bind to the resin on a magnetic stirrer at 25°C for 3 h. The resin was washed with 100 + 30 ml of washing buffer (Invitrogen). The protein was then eluted with elution buffer (50 mM NaH_2_PO_4_, 500 mM NaCl, 250 mM imidazole, pH 8.0).

The purified DmBCA recombinant protein was transferred to a buffer of 50 mM Tris-HCl, pH 7.5, using an Amicon Ultracel™ - 10 k centrifugal filter device (Millipore) according to the manufacturer's instructions. To remove the His tag, the recombinant protein was treated with 60 μl of resin-coupled thrombin (Thrombin CleanCleave KIT™, Sigma) per 1 mg of protein with gentle shaking at 25°C for 1 h, according to the manufacturer's instructions. Protein concentration was determined using the DC Protein Assay™ (Bio-Rad) with three different dilutions. Purified recombinant DmBCA proteins were analyzed using 10% sodium dodecyl sulfate polyacrylamide gel electrophoresis (SDS-PAGE) under reducing conditions. The gels were stained using the Colloidal Blue Staining Kit™ (Invitrogen).

### CA activity measurements

An Applied Photophysics stopped-flow instrument was used to assay the CA-catalyzed CO_2 _hydration activity. Phenol red (at a concentration of 0.2 mM) was used as an indicator, working at the absorbance maximum of 557 nm, with 10 - 20 mM HEPES (pH 7.5) or Tris(pH 8.3) as buffers and 20 mM Na_2_SO_4 _or 20 mM NaClO_4 _(for maintaining constant ionic strength), following the initial rates of the CA-catalyzed CO_2 _hydration reaction for a period of 10 - 100 s. The CO_2 _concentrations ranged from 1.7 to 17 mM for the determination of kinetic parameters and inhibition constants. For each inhibitor at least six traces of the initial 5-10% of the reaction were used to determine the initial velocity. The uncatalyzed rates were determined in the same manner and subtracted from the total observed rates. Stock solutions of inhibitor (100 mM) were prepared in distilled-deionized water, and dilutions up to 0.01 μM were made thereafter with distilled-deionized water. Inhibitor and enzyme solutions were preincubated together for 15 min at room temperature prior to the assay to allow for the formation of the E-I complex. The inhibition constants were obtained by non-linear least-squares methods using PRISM 3, whereas the kinetic parameters for the uninhibited enzymes were obtained from Lineweaver-Burk plots, each representing the mean of at least three different determinations.

Kinetic measurements have been performed also with m-cresol purple (as indicator) - bicine (as buffer) (data not shown), and the results were the same (±5-10% of the reported values, which is the error range of this method) both for the kinetic parameters of CO_2 _hydration and for the inhibition constants of anionic inhibitors investigated here. Thus, the standard method reported in this paper is reliable for the investigation of β-CAs (in addition to the α-CAs) as reported by this group for several enzymes, such as the three β-class enzymes from *Mycobacterium tuberculosis*, *Helicobacter pylori *and *Brucella suis*, and the fungal class enzymes (for example [25]).

### Dynamic light scattering analysis

The hydrodynamic diameters of the proteins were determined by dynamic light scattering (DLS) using Zetasizer ZS (Malvern Instruments Ltd., Worcestershire, United Kingdom). A 100 μl sample of DmBCA (200 μg/ml) in elution buffer (50 mM NaH_2_PO_4_, 500 mM NaCl, 250 mM imidazole, pH 8.0) was analyzed. Human CA II (HCA II) (180 μg/ml) in 0.1 M Tris, 0.4 M NaN_3_, 1 mM benzamidine, 20% glycerol, pH 7.0 was analyzed to support the results of DmBCA analysis. The small molecules such as imidazole and glycerol of the elution buffers seemed to dominate in the DLS analysis, since most of the light scattering was from particles having diameter smaller than one nm. Therefore, proteins were exchanged to 50 mM Na_2_HPO_4 _pH 7.0 containing 100 mM NaCl using protein desalting spin columns (Pierce). DLS analysis was then performed for 100 μl sample by using temperature scanning mode where the temperature was raised from 4°C to 50°C at 3°C intervals. Sample was let to equilibrate to each measurement temperature two minutes before data acquisition. For DmBCA three parallel measurements were carried out at each temperature and for HCA II only one measurement was performed at each temperature. The molecular weight of the protein was estimated from hydrodynamic diameter using globular protein standard curve provided by the manufacturer.

### Analytical gel filtration

The molecular size of the protein in solution was measured by size-exclusion chromatography using Superdex200 10/300GL column (GE Healthcare) connected to ÄKTA ™purifier-100 equipped with UV-900 monitor (GE Healthcare). The analysis was done using 50 mM Na_2_HPO_4_, 650 mM NaCl (pH 7.0) as mobile phase. 20-30 μg protein was injected per run. All the analyses were done with flow rate 0.3 ml/min, and absorbencies at 280 nm and 205 nm were used to locate the protein peaks in the chromatograms. Molecular weight calibration curve was prepared by analyzing gel filtration standard protein mixture containing thyroglobulin (670kDa), γ-globulin (158kDa), ovalbumin (44kDa), myoglobin (17kDa) and vitamin B_12 _(1,35kDa) (Bio-Rad).

## Authors' contributions

LS carried out protein construct design, protein production and purification, colocalization studies and drafted the manuscript. MH contributed to protein construct design and co-localization studies. MT made all the bioinformatic analysis and helped to draft the manuscript. AO contributed in bioinformatic analyses. CTS, AI and AS carried out the kinetic measurements. JL and VPH carried out the DLS analysis. BN and VPH performed the gel filtration studies. TAG conceived the study, provided materials and helped to draft the manuscript. CTS and SP conceived the study, and participated in its design and coordination and helped to draft the manuscript. All authors read and approved the final manuscript.

## Supplementary Material

Additional file 1 **The full alignment of the 26 invertebrate β-CA sequences (partly shown in Figure **1**)**. Click here for file

Additional file 2 **The full sequence alignment of all of the identified invertebrate β-CA sequences**. Click here for file
