# Supplementary material for: Characterization of the first beta-class carbonic anhydrase from an arthropod (Drosophila melanogaster) and phylogenetic analysis of beta-class carbonic anhydrases in invertebrates
Source: BMC Biochem. 2010 Jul 26;11:28. doi: 10.1186/1471-2091-11-28 (PMC2918522; doi:10.1186/1471-2091-11-28)
Supplement: Additional file 1 — The full alignment of the 26 invertebrate β-CA sequences (partly shown in Figure 1). [file 1471-2091-11-28-S1.PDF]

```
                   1
A_aegypti          ---MERILRG VMRY--RNTT REQMVKEFKQ VRDNPQPKAV FFTCMDSRMI PTRYTDTHVG
C_quinquefasciatus ---MDRILRG VMRY--RNTT REQMVKEFQK VRDNPQPKAV FFTCMDSRMI PTRYTDTHVG
A_gambiae          ---MERILRG VMRY--RHTT REQMVQEFRK VRDNPQPKAV FFTCMDSRMI PTRFTETHVG
D_melanogaster     ---MERILRG IMRY--RNTT REQMVKEFQK VRDNPEPKAV FFTCMDSRMI PTRYTDTHVG
D_virilis          ---MERILRG IMRY--RNTT REQMVKEFQK VRDNPEPKAV FFTCMDSRMI PTRYTDTHVG
T_castaneum        ---MDRILKG IMRY--RNVK KEKMVKQFKE VKDNPMPKAV FFTCIDSRMI PTRFTQTNVG
N_vitripennis      ---MDRILKG VMKY--RKCH REGMVKQFQQ VRDHPEPQAV FFTCMDSRMI PTRFTETNVG
A_mellifera        ---------- -MKY--RKCH REEMVKQFQK VKDCPEPKAA FFTCMDSRMI PTRFTETNVG
A_pisum            ---MDRIFRG IMKY--RRTN RGKMVEQFVQ VKNHPEPKAL FFTCMDSRML PARFTESNVG
D_pulex            ---MDKILKG ILKY--RKTY RTEMVEQFKQ VADRPEPKAV FFTCMDSRML PTRFTQTDVG
H_medicinalis      MPGLDKLLKG ILLY--RSTI QPHVVKQFQQ VKDNPQPKCV MFSCMDSRLV ITKMINQDVG
S_kowalevskii      ---MEKLLRG VLRY--RNGV RQELVPQFER VRDNPEPTAI LFTCMDSRML PTRFCQTNVG
X_bocki            MPGMEKLLNG IMKF--RGTI RNDLVQQFQR VKDNPNPTCL FFTCIDSRML PSRFTQTNVG
C_elegans_1        ---MNKILRG VIQF--RNTI RKDLVKQFEE IKNNPSPTAV MFTCMDSRML PTRFTQSQVG
P_pacificus_1      ---MQRILRG VIQY--RQTV RKELVEQFKE IKDNPQPKAL MFTCMDSRML PTRFTQAKVG
A_caninum          MPGLHKVLQG IVKF--RQTA RKELVKQFEQ IRNNPHPTAV FFSCMDSRML PARFTSSQVG
C_elegans_2        MPGLERILNG VIRF--RQTV RKDLVKQFER IRDNPHPTAV FFTCMDSRML PARITSSQVG
P_pacificus_2      MPGLNKILNG VIRF--RQTV RKDLVKQFEK IRDNPHPTAV FFTCMDSRML PARFTQSQVG
T_adhaerens        --MIEKVIRG VLRY--SASL SSANKTLYAQ VAEKVQPSCI FITCMDSRVF PSNIASIAPG
C_clemensi         ---MDKVLRG ILQYNRSAKK KDVLKQLSKI VDSQSTPSSV LFTCMDSRIH PNVFMNSDIG
L_salmonis         ---MEKVFRG IIRY-KNAYK DDVFTKLSKI KESGSSPSTV LFTCMDARIH PNVIMNSNVG
M_senile           ---MDRILRG IVKF--RNSL RPSLLPLLKN VAK---PDML LVTCVDSRLL PCSYTSAVPG
N_vectensis        ---MEKILQG VVRF--RHVL RPSLLPSLRE VAEKVAPKTV LVACVDCRIM PETYMSSEPG
A_pectinifera      ---MEKILRG IIKY--KASN -FLVRWATHA PKQHVKPPIL FVSCVDSRVL PTHFCQTSPG
P_lividus          ---MRKVLVQ VSRR--FSSQ PGVTAKTRPF QVGLKPPLAV LVTCMDGRLL PSRIFKADRG
S_purpuratus       ---------- ---------- ---------- ------PLAV LVTCMDGRLL PSRIFKAERG

                  61
A_aegypti          DMFVIRNAGN LVPHAEHFQD -------EYF SCEPAGLELG CVVNNIKHII VCGHSDCKAM
C_quinquefasciatus DMFVVRNAGN LVPHAEHFQD -------EYF SCEPAALELG CVVNNIKHII VCGHSDCKAM
A_gambiae          DMFVVRNAGN LVPHAEHFQD -------EYF SCEPAALELG CVVNNIKHII VCGHSDCKAM
D_melanogaster     DMFVVRNAGN LIPHAQHFQD -------EYF SCEPAALELG CVVNDIRHII VCGHSDCKAM
D_virilis          DMFVVRNAGN LIPHAQHFQD -------EYF SCEPAALELG CVVNDIRHII VCGHSDCKAM
T_castaneum        DMFVVRNAGN IIPHSQHFLD -------ELT TNEPAALELG CVVNDIRHII VCGHSDCKAI
N_vitripennis      DMFVVRNAGN IVPHSQHFID -------ELT MCEPAALELG CVVNDIRHVI VCGHSDCKAM
A_mellifera        DMFVVRNAGN IIPHSQHFED -------ELA MCEPAALELV CLMNEIKHII VCGHSDCKAM
A_pisum            DMFIVRNAGN LIPHSQHFPD -------EYT SCEPAALELG CVHNDIRHVI VCGHSDCKAM
D_pulex            DMFIVRNAGN LVPHSKLYGI -------DSA TTEPAALELG CIVNNVKHMV VCGHSDCKAM
H_medicinalis      DMFLVRNAGN LIPNNDSLSF -------DSV TTEPAALELG CIINNIRHVV VCGHSDCKAM
S_kowalevskii      DMFMVRNAGN LIPHSELFCG -------DSL NTEPAALELA CIKNDVNHVI VCGHSDCKAM
X_bocki            DMYIVRNVGN VIPHSHMYDR LH---EHGIV TTEPAAMDLA CVTGSIRHVV VCGHSDCLAM
C_elegans_1        DMFVVRNAGN MIPDAPNYG- AF---SEVSV NTEPAALELA VKRGGIRHIV VCGHSDCKAI
P_pacificus_1      DIFVVRNAGN LIPDACNYG- HY---SEVSC TTEPAALELA VKRGGVKHVI VCGHSDCKAM
A_caninum          DMFVVRNSGN MIPHANNYGP AG---YEVSV TTEPAALELA VKRGHINHVI VCGHSDCKAI
C_elegans_2        DMFVVRNSGN MIPHANNYGP SG---YEVSV TTEPAALELA VKRGKINHVI VCGHSDCKAI
P_pacificus_2      DMFVVRNSGN MIPHANNYGP VG---YEVSV TTEPAALELA VKRGKINHVI VCGHSDCKAI
T_adhaerens        ESFIVRNAGN IVPHSKLIYE ------RWTP A-EAAALELA CVRNQVSSVV VCGHSDCKAM
C_clemensi         DAFTVRNAGN IVPKSGLVHG ------LVNP APEPAGLELG CVLNSIKNVI VCGHSDCKAM
L_salmonis         DVFTVRNPGN IVPNASYVAN ------SRTP APEPAGLELG CVVNSIKNVV VCGHFDCKAM
M_senile           QMFVVRNVGN LFPHARLFGS ------QVSA TAEAAALELA VVNYGIKHVA VCGHSDCKAM
N_vectensis        DMFVVRTAGN LLPHAKLYG- ------DVGS CSELAALQMA IQEGKVENVV VCGHSNCKGM
A_pectinifera      DMFILRNAGN VIPRANYSEG E-----PTHI SCEVVALEIT CNRSKVEHVV VCGHSDCQAL
P_lividus          ELLIIRNPGN FVPHSCKCEP LEGSSAPVYP SGEMAGLQLA IQKMAIPDVI VCGHTDCRAG
S_purpuratus       ELLIIRNPGN FVPHSCKCEP SEGSEAPAFP SGELAGLQLA IQKMAIPDVI VCGHTDCRAG
```

```
                   121
A_aegypti          NLLYQLRDPE FSSRKNRRIS ----PLRAWL CEHANTS-LE KFQNLRQVGL DK-PLIFSSE
C_quinquefasciatus NLLYQLRDPQ FASRKNRRIS ----PLRAWL CEHADTS-LE KFQNLQETGL DK-PIIFSSE
A_gambiae          NLLYKLKDPE FASLDNRRIS ----PLRAWL CEHANTS-LA KFQNLKEIGL DK-PLIFSSE
D_melanogaster     NLLYQLRDPD FASKLNRRLS ----PLRSWL CTHANTS-LE RFQEWRDAGM KD-PLIFSSE
D_virilis          NLLYQLRDPD FASKLNRRLS ----PLRSWM CTHANTS-LE KFQEWRDAGM KD-PLIFSSE
T_castaneum        NLLYKLQDSE FASQDNRRIS ----PLRAWL CTHALTS-LE KFQQLQVTDY GK-PLIFQAE
N_vitripennis      NLLYALRDEE FASQVNRRIS ----PLRAWL CAHGSSS-LA KFQQLEITGF HE-PLLFQAE
A_mellifera        NMLYSLREEE LASKVNRRIS ----PLKAWL CAHASNS-LT RFQQLEISDF RD-PILFQGE
A_pisum            NLLHLLRDTE YGSTVNRRKS ----PLRAWL CSHAMSS-LE KYQQLEAAGF GT-PLVFQAE
D_pulex            NLLYSFKKGI ETNMRTLERS ----PLKAWL HRHGSIS-LT KFERLEVHGF QQ-PLTFPME
H_medicinalis      NALYGMMD-- --SVQKHEGT ----PLQIWL KRHGART-LV KYKELLQAGG VG-PIKFQAE
S_kowalevskii      NCLYGIRN-- -VTSHDKDKN ----PFAAWL AKFGKAS-LN AFKEVERG-S KS-PLKFTGE
X_bocki            KTLSGIHD-- -ECCVESKDT ----HIQTFL RKNGQST-ME KFDLHKQS-M LS-SLTFQGE
C_elegans_1        NTLYGLHQ-- -CPKNFDVTS ----PMDHWV RRNGFAS-VK RLNERLHRGP S--SMKFESE
P_pacificus_1      NMLFGLHA-- -CPSNFDHAS ----PMDHWL RKNGHRT-MK KLNERLYKGP Q--PLQFDSE
A_caninum          NTLYNIHE-- -CPHTFDPQS ----PMDHWL RRHGFAS-LK KLEERLADKT AK-PIKFVSD
C_elegans_2        NTLYNLHK-- -CPKSFDPES ----PMDHWL RRHGFNS-IR KLEKRLADKN AG-PIEFVSD
P_pacificus_2      NTLYNLHC-- -CPKSFDPES ----PMDHWL RRHGYNS-LR KLEKRLADDK AG-PLEFVSS
T_adhaerens        DGLHSLG--G TAPSE----S S---FVLDWI YRFASQT-YT KWEKTTLVDR SNSDQPLHLE
C_clemensi         IAVHSLKD-S NGWSEEELLQ S---PLKAWL YKHGMDS-LN KLND-KLTSP ESP-----LT
L_salmonis         IALQSFGD-S KGCSEFDVMQ S---PLKAWL QRNGMVS-FK RFCEMKKMGK EDS-----LI
M_senile           HALYDTTTCS KTDNNS---- ----MITSWI TTHGKSS-LT KLDQLIVNKA KPVKLVFNE-
N_vectensis        TFLLSHD--S RTDN------ ----HYIPWL KKTGASS-LT RFEKVDMSQE GGVKLLFED-
A_pectinifera      YAAYSHYTEK ETKKPLKRVS Q---VIHQWC RTHGVEASLN KIQDLSARTP SEGVMTFDVG
P_lividus          EALRNLPISK PTGNVGSGSQ HSLDLTNNWL HAYGSPA-LE KYQKHMENPE EEVRFEGGGR
S_purpuratus       EALRHLPVSR PTGQTGSGSQ HSMDLMNNWL RAYGSPA-LE KYERHMENPA EEVTYEGGGR

                   181
A_aegypti          -TPLRKFVAY IDPENQFALE DKLSQVNTLQ QIENVASYGF LKKRLESHDL HIHALWFDIY
C_quinquefasciatus -TPLRKFVAY IDPENQFAIE DKLSQVNTLQ QIENIASYGF LKKRLESHDL HIHALWFDIY
A_gambiae          -TPLRKFVAY IDPENNFAIE DKLSQVNTLQ QIENVASYGF LKRRLESHDL HIHALWFDIY
D_melanogaster     -TPLRRFVAY IDEEQKFALE DKLSQINTLQ QMSNIASYGF LKARLESHDL HIHALWFDIY
D_virilis          -TPLSRFVAY IDEENKFAIE DKLSQINTLQ QMSNVASYGF LKTRLETHNL HVHALWFDIY
T_castaneum        -TPMRKFVAY IDPENKFTIE DKLSQINTLQ QLQNIASYGF LKKRLEKHQL HIHALWFDIY
N_vitripennis      -TPLRKFVAY IDPEDKFAIE DKLSQINTLQ QLQNVASYGF LKRRLEKHNL HVHALWFDIY
A_mellifera        -TSLRKFVAY IDPEDKFGVE DKLSQINTLQ QLQNIASYGF LKKRLERHDL HIHALWFDIY
A_pisum            -TPLRRISAY IDPEDKLSVT DKLSQVNTLQ QIQNIASYDF LKKRLETYDL HIHALWFDIY
D_pulex            -GPFRQFVAY IDPDNKFSLT DKLSQLNTLQ QLQHIASYSF IQSAINSGRV HLHALWFDIY
H_medicinalis      -TPEKIFDAY IDVENQFKPV DKLSQVNTLQ QLQNIASHPF LKKKLEIGKV RLHALWIDVY
S_kowalevskii      -TPKHNFEAF IDPDDKFGIE DKLSQVNCLQ QLQNISSYGF LKDRLESGRV RLHAMW----
X_bocki            -TPRHNFEAY IDPDDKFQMV DKLSQINCLQ QLQNISSWGF LRSKLENNEV RLHAMWFDIY
C_elegans_1        VAPSQSFDAI IDPMDTLAME DKLSQINVLQ QLINICSHEF LKEYLESGRL HIHGMWFDIY
P_pacificus_1      VAPSQSFEAI IDPFDRLKAE DKLSQINVLQ QLVNIASHDA LKEAFDQGKL HIHGMWFDVY
A_caninum          -NPSFSFEAV IDPEDKWGVE DKLSQINTLQ QLENCASHGF LTEFLEKKTV DLHAMWFDIF
C_elegans_2        -NPLFSFSAV IDPEDKLNVE DKLSQINTLQ QLENVASHGF LKEFLVSQTV DLHAMWFDIY
P_pacificus_2      -NPLFSFSAI IDAEGKWNVE DRLSQINTLQ QLENIASHGF LNEFLESRQV DLHAMWFDVY
T_adhaerens        FNEN-GLKFE ANINQNLLPK DQLSQINTLQ QLLHVNSYSF MKEKIAAGTV KLYSLWFDIK
C_clemensi         FMKDTQHEFE ANMDNKLLES DQLSQINTLV QIENIYSYGF MKERMDQHQS VAHGLWLSLS
L_salmonis         FMKNTKHEFE ARIDSQLDEA DQLSQINTLV QMENIYSYDF MKNRIDDKTA FVHGLWFSLT
M_senile           DNDAERFEAT IDEN--LNIQ DKLSQVNVLQ QLHNIGSYSF VREQLRAKQL NLLALWFDTA
N_vectensis        ATGGEPMEVT IDEGNKLDSV DKLSQVNVLQ QLHNLKSFPF ISNPLSKGAL NLYGLWFDIK
A_pectinifera      ATPIEAYFKD FDSKDEKSLL DKLSKVNVLQ QLEHLSSYRS IRRRVIAGTL SLHGMWYDVA
P_lividus          -KGVKMAAVI EDPDSKLSNT DRLAQINVLQ QLEHLQSYDF IGKRMETDQI RLHATFYDTV
S_purpuratus       -KGAKLSAVI ED-NGKLSKT DRLAQINVLQ QLEHLQSYDF IGKRMETDQI RLHATFYDTF
```

```
                    241
A_aegypti           TGDIYYFSRN SKRFIPIDET SIEQLLDEVR RYYS------ ----------
C_quinquefasciatus  TGDIYYFSRN SKRFIPVDET TIERLLKEVN QFYS------ ----------
A_gambiae           TGDIYFFSRN SKRFIAIDES SIDRLLDEVR RYYS------ ----------
D_melanogaster      TGDIYYFSRG AKRFLPVDED TVDRLSEEVR RFYS------ ----------
D_virilis           TGDIYYFSRG AKRFIAVDES SVDQLSAEVR RFYS------ ----------
T_castaneum         TGEIYYFSRG AKKFVVIDEE NFPKLLQEVE KYYS------ ----------
N_vitripennis       TGDIYYFSRA NKRFVEINEL TETPLLKEIK KYYS------ ----------
A_mellifera         TGDIYYFSRA NKKFVEINES TERCLLTEIK KYYS------ ----------
A_pisum             TGDVHYFSRQ SKQFVEINEK NVDGLVEEVS KYYC------ ----------
D_pulex             TGDIYVFSRK QKRFVEISEE TSDYLLDEIR EYFV------ ----------
H_medicinalis       TGDFHMFCRD SNRFMLVNEE SYENLLADGE SNIDY----- ----------
S_kowalevskii       ---------- ---------- ---------- ---------- ----------
X_bocki             TGDHYMFQQN TGNGLSKSAK KTWSTY---- ---------- ----------
C_elegans_1         KGEDYLFSKD KKRFVVIDEK TVTDLLAELN ARYPVPEDQD GPVAFAKSN
P_pacificus_1       KGEDYLFSKE KRQFVII--- ---------- ---------- ----------
A_caninum           AGEMYLFSKP RRKFILIDEG TVDKLEEEVN QHKA------ ----------
C_elegans_2         TGEMHMFSKP NKQFVLVDES NVEELIDEVE KHQT------ ----------
P_pacificus_2       AGEMYMFSKP RKQ------- ---------- ---------- ----------
T_adhaerens         DATCYVFNKR DKLFQPIVSE EMLKSVSEFQ INQK------ ----------
C_clemensi          SGEAHFFSKK DKAFVNVTED NVEELVCR-- ---------- ----------
L_salmonis          TGDVHYFLKN EKVFINVSED NIDNLVQRSE E--------- ----------
M_senile            NGEMYVFSKQ SGTFELITDN TLL------- ---------- ----------
N_vectensis         EGEMYMFSRK QKKFVLINKD TVNNLCSEVD ---------- ----------
A_pectinifera       TDKIYWFSRA RRMFLEINE- ---------- ---------- ----------
P_lividus           TGHMSVFSQS QGRFNFLPTE DFSSLSHYIF ELKSS----- ----------
S_purpuratus        SGNVYVFNQK QGRFNFLPTA DIDSLSHYIF QLRSS----- ----------
```
