# Supplementary material for: Characterization of the first beta-class carbonic anhydrase from an arthropod (Drosophila melanogaster) and phylogenetic analysis of beta-class carbonic anhydrases in invertebrates
Source: BMC Biochem. 2010 Jul 26;11:28. doi: 10.1186/1471-2091-11-28 (PMC2918522; doi:10.1186/1471-2091-11-28)
Supplement: Additional file 2 — The full sequence alignment of all of the identified invertebrate β-CA sequences. [file 1471-2091-11-28-S2.PDF]

```
                                1
S_kowalevskii_187043763         ----MEKLLR GVLRY---RN GVRQELVPQF ERVRDN-PEP TAILFTCMDS
X_bocki_117195962               -MPGMEKLLN GIMKF---RG TIRNDLVQQF QRVKDN-PNP TCLFFTCIDS
D_pseudoobscura_125776717       ----MERILR GVMRY---RN TTREQMVKEF QKVRDN-PEP KAVFFTCMDS
D_willistoni_194166182          ----MERILR GIMRY---RN TTREQMVKEF QKVRDN-PEP KAVFFTCMDS
D_melanogaster_24645213         ----MERILR GIMRY---RN TTREQMVKEF QKVRDN-PEP KAVFFTCMDS
D_erecta_190652591              ----MERILR GIMRY---RN TTREQMVKEF QKVRDN-PEP KAVFFTCMDS
D_virilis_194152748             ----MERILR GIMRY---RN TTREQMVKEF QKVRDN-PEP KAVFFTCMDS
D_mojavensis_193916036          ----MERILR GIMRY---RN TTREQMVKEF QKVRDH-PEP KAVFFTCMDS
D_grimshawi_193894023           ----MERILR GVMRY---RN TTREQMVKEF QKVRDN-PEP KAVFFTCMDS
D_ananassae_190626360           ----MERILR GIMRY---RN TTREQMVKEF QKVRDN-PEP KAVFFTCMDS
A_aegypti_157110803+77891004    ----MERILR GVMRY---RN TTREQMVKEF KQVRDN-PQP KAVFFTCMDS
A_gambiae_57968460              ----MERILR GVMRY---RH TTREQMVQEF RKVRDN-PQP KAVFFTCMDS
C_quinquefasciatus_170043321    ----MDRILR GVMRY---RN TTREQMVKEF QKVRDN-PQP KAVFFTCMDS
T_castaneum_91084165            ----MDRILK GIMRY---RN VKKEKMVKQF KEVKDN-PMP KAVFFTCIDS
N_vitripennis_156547528         ----MDRILK GVMKY---RK CHREGMVKQF QQVRDH-PEP QAVFFTCMDS
A_mellifera_110764310           ---------- --MKY---RK CHREEMVKQF QKVKDC-PEP KAAFFTCMDS
A_pisum_193713675               ----MDRIFR GIMKY---RR TNRGKMVEQF VQVKNH-PEP KALFFTCMDS
D_pulex_FE417346+FE409868       ----MDKILK GILKY---RK TYRTEMVEQF KQVADR-PEP KAVFFTCMDS
H_medicinalis_EY481200+EY50505  -MPGLDKLLK GILLY---RS TIQPHVVKQF QQVKDN-PQP KCVMFSCMDS
C_elegans_NP_741809.1           ----MNKILR GVIQF---RN TIRKDLVKQF EEIKNN-PSP TAVMFTCMDS
C_brennerii_BCA1                ----MNKILR GVIQF---RN TIRKDLVKQF EEVKNN-PSP TAVMFTCMDS
C_japonica_BCA1                 ----MNKILR GVIKF---RQ TIREDLVKQF EDIKNN-PKP TAVMFTCMDS
C_remanei_BCA1                  ----MNKILR GVIKY---RQ TIREDLVKQF EEIKNN-PQP TSVMFTCMDS
C_briggsae_A8XKV0               ----MNRIIR GVIQY---NQ KIKAGLVKQF EHVSDH-PNP TAVMFTCMDS
P_pacificus_BCA1                ----MQRILR GVIQY---RQ TVRKELVEQF KEIKDN-PQP KALMFTCMDS
C_briggsae_BCA2                 -MPGLQKILN GVIRF---RQ TVRKDLVKQF EQVRDN-PHP TAVFFTCMDS
C_remanei_BCA2                  -MPGLQKILN GVIRF---RQ TVRKDLVKQF EQIRDN-PHP TAVFFTCMDS
C_brennerii_BCA2                -MPGLQKILN GVIRF---RQ TVRKDLVKQF EHIRDN-PHP TAVFFTCMDS
C_elegans_NP_001041015          -MPGLERILN GVIRF---RQ TVRKDLVKQF ERIRDN-PHP TAVFFTCMDS
P_pacificus_BCA2                -MPGLNKILN GVIRF---RQ TVRKDLVKQF EKIRDN-PHP TAVFFTCMDS
A_caninum_FC551456+FC550353     -MPGLHKVLQ GIVKF---RQ TARKELVKQF EQIRNN-PHP TAVFFSCMDS
B_floridae_210100262            ---------- ---------- ---------- ---------- ----------
B_floridae_210094934            ---------- ---------- ---------- ---------- ----------
M_senile_FC835283               ----MDRILR GIVKF---RN SLRPSLLPLL KNVAK----P DMLLVTCVDS
N_vectensis_XP_001632619        ----MEKILQ GVVRF---RH VLRPSLLPSL REVAEK-VAP KTVLVACVDC
A_pectinifera_DB424979+DB44052  ----MEKILR GIIKY---KA SN-FLVRWAT HAPKQH-VKP PILFVSCVDS
C_clemensi_225719368            ----MDKVLR GILQYN--RS AKKKDVLKQL SKIVDSQSTP SSVLFTCMDS
L_salmonis_225713548            ----MEKVFR GIIRY---KN AYKDDVFTKL SKIKESGSSP STVLFTCMDA
T_adhaerens_190581916           ---MIEKVIR GVLRY---SA SLSSANKTLY AQVAEK-VQP SCIFITCMDS
P_lividus_139313180+139245724   MQNGGSRLMR KVLVQVSRRF SSQPGVTAKT RPFQVGLKPP LAVLVTCMDG
S_purpuratus_XP_001189115       ---------- ---------- ---------- ---------P LAVLVTCMDG
```

```
                                  51
S_kowalevskii_187043763           RMLPTRFCQT NVGDMFMVRN AGNLIPHSEL FCG------- DSLNTEPAAL
X_bocki_117195962                 RMLPSRFTQT NVGDMYIVRN VGNVIPHSHM YDRLH---EH GIVTTEPAAM
D_pseudoobscura_125776717         RMIPTRYTDT HVGDMFVVRN AGNLIPHAQH FQD------- EYFSCEPAAL
D_willistoni_194166182            RMIPTRYTDT HVGDMFVVRN AGNLIPHAQH FQD------- EYFSCEPAAL
D_melanogaster_24645213           RMIPTRYTDT HVGDMFVVRN AGNLIPHAQH FQD------- EYFSCEPAAL
D_erecta_190652591                RMIPTRYTDT HVGDMFVVRN AGNLIPHAQH FQD------- EYFSCEPAAL
D_virilis_194152748               RMIPTRYTDT HVGDMFVVRN AGNLIPHAQH FQD------- EYFSCEPAAL
D_mojavensis_193916036            RMIPTRYTDT HVGDMFVVRN AGNLIPHAQH FQD------- EYFSCEPAAL
D_grimshawi_193894023             RMIPTRYTDT HVGDMFVVRN AGNLIPHAHH FHD------- EYFSCEPAAL
D_ananassae_190626360             RMIPTRYTDT HVGDMFVVRN AGNLIPHAHH FQD------- EYFSCEPAAL
A_aegypti_157110803+77891004      RMIPTRYTDT HVGDMFVIRN AGNLVPHAEH FQD------- EYFSCEPAGL
A_gambiae_57968460                RMIPTRFTET HVGDMFVVRN AGNLVPHAEH FQD------- EYFSCEPAAL
C_quinquefasciatus_170043321      RMIPTRYTDT HVGDMFVVRN AGNLVPHAEH FQD------- EYFSCEPAAL
T_castaneum_91084165              RMIPTRFTQT NVGDMFVVRN AGNIIPHSQH FLD------- ELTTNEPAAL
N_vitripennis_156547528           RMIPTRFTET NVGDMFVVRN AGNIVPHSQH FID------- ELTMCEPAAL
A_mellifera_110764310             RMIPTRFTET NVGDMFVVRN AGNIIPHSQH FED------- ELAMCEPAAL
A_pisum_193713675                 RMLPARFTES NVGDMFIVRN AGNLIPHSQH FPD------- EYTSCEPAAL
D_pulex_FE417346+FE409868         RMLPTRFTQT DVGDMFIVRN AGNLVPHSKL YGI------- DSATTEPAAL
H_medicinalis_EY481200+EY50505    RLVITKMINQ DVGDMFLVRN AGNLIPNNDS LSF------- DSVTTEPAAL
C_elegans_NP_741809.1             RMLPTRFTQS QVGDMFVVRN AGNMIPDAPN YG-AF---SE VSVNTEPAAL
C_brennerii_BCA1                  RMLPTRFTQS QVGDMFVVRN AGNMIPDAPN YG-AF---SE VSVNTEPAAL
C_japonica_BCA1                   RMLPTRFTQS QVGDMFVVRN AGNMIPDAPN YG-AF---SE VSVNTDPAAL
C_remanei_BCA1                    RMLPTRFTQS RVGDMFVVRN AGNMIPEAPT YG-TS---SE VSVTTEPAAL
C_briggsae_A8XKV0                 RMLPTRFTQS AVGDMFVVRN AGNMIPAAPN YG-SY---SE VSINTEPAAL
P_pacificus_BCA1                  RMLPTRFTQA KVGDIFVVRN AGNLIPDACN YG-HY---SE VSCTTEPAAL
C_briggsae_BCA2                   RMLPARITSS QVGDMFVVRN SGNMIPHANN YGPSG---YE VSVTTEPAAL
C_remanei_BCA2                    RMLPARITSS QVGDMFVVRN SGNMIPHANN YGPSG---YE VSVTTEPAAL
C_brennerii_BCA2                  RMLPARITSS QVGDMFVVRN SGNMIPHANN YGPSG---YE VSVTTEPAAL
C_elegans_NP_001041015            RMLPARITSS QVGDMFVVRN SGNMIPHANN YGPSG---YE VSVTTEPAAL
P_pacificus_BCA2                  RMLPARFTQS QVGDMFVVRN SGNMIPHANN YGPVG---YE VSVTTEPAAL
A_caninum_FC551456+FC550353       RMLPARFTSS QVGDMFVVRN SGNMIPHANN YGPAG---YE VSVTTEPAAL
B_floridae_210100262              ---------- ---------- -GNLIPHSKL YGK------- EVLGSEVASM
B_floridae_210094934              ---------- ---------- -GNLIPHSKL YGR------- EVLGSEVASM
M_senile_FC835283                 RLLPCSYTSA VPGQMFVVRN VGNLFPHARL FGS------Q VSATAEAAAL
N_vectensis_XP_001632619          RIMPETYMSS EPGDMFVVRT AGNLLPHAKL YG-------D VGSCSELAAL
A_pectinifera_DB424979+DB44052    RVLPTHFCQT SPGDMFILRN AGNVIPRANY SEGE-----P THISCEVVAL
C_clemensi_225719368              RIHPNVFMNS DIGDAFTVRN AGNIVPKSGL VHG------L VNPAPEPAGL
L_salmonis_225713548              RIHPNVIMNS NVGDVFTVRN PGNIVPNASY VAN------S RTPAPEPAGL
T_adhaerens_190581916             RVFPSNIASI APGESFIVRN AGNIVPHSKL IYER------ -WTPAEAAAL
P_lividus_139313180+139245724     RLLPSRIFKA DRGELLIIRN PGNFVPHSCK CEPLEGSSAP VYPSGEMAGL
S_purpuratus_XP_001189115         RLLPSRIFKA ERGELLIIRN PGNFVPHSCK CEPSEGSEAP AFPSGELAGL
```

```
                                101
S_kowalevskii_187043763          ELACIKNDVN HVIVCGHSDC KAMNCLYGIR N---VTSHDK DKN----PFA
X_bocki_117195962                DLACVTGSIR HVVVCGHSDC LAMKTLSGIH D---ECCVES KDT----HIQ
D_pseudoobscura_125776717        ELGCVVNDIR HIIVCGHSDC KAMNLLYQLR DPEFASKLNR RLS----PLR
D_willistoni_194166182           ELGCVVNDIR HIIVCGHSDC KAMNLLYQLR DPEFASKLNR RLS----PLR
D_melanogaster_24645213          ELGCVVNDIR HIIVCGHSDC KAMNLLYQLR DPDFASKLNR RLS----PLR
D_erecta_190652591               ELGCVVNDIR HIIVCGHSDC KAMNLLYQLR DPDFASKLNR RLS----PLR
D_virilis_194152748              ELGCVVNDIR HIIVCGHSDC KAMNLLYQLR DPDFASKLNR RLS----PLR
D_mojavensis_193916036           ELGCVVNDIR HIIVCGHSDC KAMNLLYQLR DPDFASKLNR RLS----PLR
D_grimshawi_193894023            ELGCVVNDIR HIIVCGHSDC KAMNLLYQLR DPEFASKLNR RLS----PLR
D_ananassae_190626360            ELGCVVNDIR HIIVCGHSDC KAMNLLYQLR DPEFASKLNR RLS----PLR
A_aegypti_157110803+77891004     ELGCVVNNIK HIIVCGHSDC KAMNLLYQLR DPEFSSRKNR RIS----PLR
A_gambiae_57968460               ELGCVVNNIK HIIVCGHSDC KAMNLLYKLK DPEFASLDNR RIS----PLR
C_quinquefasciatus_170043321     ELGCVVNNIK HIIVCGHSDC KAMNLLYQLR DPQFASRKNR RIS----PLR
T_castaneum_91084165             ELGCVVNDIR HIIVCGHSDC KAINLLYKLQ DSEFASQDNR RIS----PLR
N_vitripennis_156547528          ELGCVVNDIR HVIVCGHSDC KAMNLLYALR DEEFASQVNR RIS----PLR
A_mellifera_110764310            ELVCLMNEIK HIIVCGHSDC KAMNMLYSLR EEELASKVNR RIS----PLK
A_pisum_193713675                ELGCVHNDIR HVIVCGHSDC KAMNLLHLLR DTEYGSTVNR RKS----PLR
D_pulex_FE417346+FE409868        ELGCIVNNVK HMVVCGHSDC KAMNLLYSFK KGIETNMRTL ERS----PLK
H_medicinalis_EY481200+EY50505   ELGCIINNIR HVVVCGHSDC KAMNALYGMM D----SVQKH EGT----PLQ
C_elegans_NP_741809.1            ELAVKRGGIR HIVVCGHSDC KAINTLYGLH Q---CPKNFD VTS----PMD
C_brennerii_BCA1                 ELAVKRGGIR HIVVCGHSDC KAINTLYGLH Q---CPKNFD VTS----PMD
C_japonica_BCA1                  ELAVKRGGIR HIVVCGHSDC KAINTLYGLH Q---CPKNFD VAS----PMD
C_remanei_BCA1                   ELAVKRGGIR HVVVCGHSDC KAINTLYRLH Q---CPKEFD PSS----PMD
C_briggsae_A8XKV0                ELAVKRGKIR HVVVCGHSDC KAMNTLYQLH Q---CPTKFD VSS----PMD
P_pacificus_BCA1                 ELAVKRGGVK HVIVCGHSDC KAMNMLFGLH A---CPSNFD HAS----PMD
C_briggsae_BCA2                  ELAVKRGKIN HVIVCGHSDC KAINTLYNLH K---CPKSFD PES----PMD
C_remanei_BCA2                   ELAVKRGKIN HVIVCGHSDC KAINTLYNLH K---CPKSFD AES----PMD
C_brennerii_BCA2                 ELAVKRGKIN HVIVCGHSDC KAINTLYNLH K---CPKSFD PES----PMD
C_elegans_NP_001041015           ELAVKRGKIN HVIVCGHSDC KAINTLYNLH K---CPKSFD PES----PMD
P_pacificus_BCA2                 ELAVKRGKIN HVIVCGHSDC KAINTLYNLH C---CPKSFD PES----PMD
A_caninum_FC551456+FC550353      ELAVKRGHIN HVIVCGHSDC KAINTLYNIH E---CPHTFD PQS----PMD
B_floridae_210100262             EWACSQGSLE HVIVCGHSNC QVKSILDVLG KSQIQSAPNC RSS----PFL
B_floridae_210094934             EWACSQGSLE HVIVCGHSNC Q---ILDVLG KSQIQSASDC RSS----PFL
M_senile_FC835283                ELAVVNYGIK HVAVCGHSDC KAMHALYDTT TCSKTDNNS- -------MIT
N_vectensis_XP_001632619         QMAIQEGKVE NVVVCGHSNC KGMTFLLSHD --SRTDN--- -------HYI
A_pectinifera_DB424979+DB44052   EITCNRSKVE HVVVCGHSDC QALYAAYSHY TEKETKKPLK RVSQ---VIH
C_clemensi_225719368             ELGCVLNSIK NVIVCGHSDC KAMIAVHSLK DSNGWSEEEL LQS----PLK
L_salmonis_225713548             ELGCVVNSIK NVVVCGHFDC KAMIALQSFG DSKGCSEFDV MQS----PLK
T_adhaerens_190581916            ELACVRNQVS SVVVCGHSDC KAMDGLHSLG G------TAP SESS---FVL
P_lividus_139313180+139245724    QLAIQKMAIP DVIVCGHTDC RAGEALRNLP ISKPTGNVGS GSQHSLDLTN
S_purpuratus_XP_001189115        QLAIQKMAIP DVIVCGHTDC RAGEALRHLP VSRPTGQTGS GSQHSMDLMN
```

```
                              151
S_kowalevskii_187043763         AWLAKFGKAS -LNAFKEVER G-SKS-PLKF TGET--PKHN FEAFIDPDDK
X_bocki_117195962               TFLRKNGQST -MEKFDLHKQ S-MLS-SLTF QGET--PRHN FEAYIDPDDK
D_pseudoobscura_125776717       SWLCTHANTS -LEKFQEWHD AGMKD-PIFF SSET--PLRR FVAYIDSEKK
D_willistoni_194166182          SWLCTHANTS -LEKFQEWRD AGMND-PLLF SSES--PLRR FVAYIDKDQK
D_melanogaster_24645213         SWLCTHANTS -LERFQEWRD AGMKD-PLIF SSET--PLRR FVAYIDEEQK
D_erecta_190652591              SWLCTHANTS -LERFQEWRD AGMKD-PLIF SSET--PLRR FVAYIDEEQK
D_virilis_194152748             SWMCTHANTS -LEKFQEWRD AGMKD-PLIF SSET--PLSR FVAYIDEENK
D_mojavensis_193916036          SWMCTHANSS -LEKFQEWRD AGMKD-PLIF SSET--PLRR FVAYIDEEQK
D_grimshawi_193894023           SWLCTHANTS -LDRFQEWRD AGMKD-ALVF SSET--PLRR FVAYIDQDDK
D_ananassae_190626360           SWMCTHASTS -LEKFQEWRD AGMKD-PLLF SSET--PLRR FVAYIDEEQK
A_aegypti_157110803+77891004    AWLCEHANTS -LEKFQNLRQ VGLDK-PLIF SSET--PLRK FVAYIDPENQ
A_gambiae_57968460              AWLCEHANTS -LAKFQNLKE IGLDK-PLIF SSET--PLRK FVAYIDPENN
C_quinquefasciatus_170043321    AWLCEHADTS -LEKFQNLQE TGLDK-PIIF SSET--PLRK FVAYIDPENQ
T_castaneum_91084165            AWLCTHALTS -LEKFQQLQV TDYGK-PLIF QAET--PMRK FVAYIDPENK
N_vitripennis_156547528         AWLCAHGSSS -LAKFQQLEI TGFHE-PLLF QAET--PLRK FVAYIDPEDK
A_mellifera_110764310           AWLCAHASNS -LTRFQQLEI SDFRD-PILF QGET--SLRK FVAYIDPEDK
A_pisum_193713675               AWLCSHAMSS -LEKYQQLEA AGFGT-PLVF QAET--PLRR ISAYIDPEDK
D_pulex_FE417346+FE409868       AWLHRHGSIS -LTKFERLEV HGFQQ-PLTF PMEG--PFRQ FVAYIDPDNK
H_medicinalis_EY481200+EY50505  IWLKRHGART -LVKYKELLQ AGGVG-PIKF QAET--PEKI FDAYIDVENQ
C_elegans_NP_741809.1           HWVRRNGFAS -VKRLNERLH RGPS--SMKF ESEVA-PSQS FDAIIDPMDT
C_brennerii_BCA1                HWVRRNGFAS -VKRLNERLH LGPS--NMSF ESEVS-PSQS FEAIIDPMDR
C_japonica_BCA1                 HWVRRNGFNS -VKRLNERLH RGPS--LMKY DSEVS-PSQS FEAMIDPMDK
C_remanei_BCA1                  NWVRRSGYSS -IKRLNERIH RGPS--IMKF DSEVA-PSQS FEAIIDPMDK
C_briggsae_A8XKV0               QWLRRNGFES -MKKLNERLH IGPK--TMKF ESEVA-PSQS FEAIIDPMEK
P_pacificus_BCA1                HWLRKNGHRT -MKKLNERLY KGPQ--PLQF DSEVA-PSQS FEAIIDPFDR
C_briggsae_BCA2                 HWLRRHGFNS -IKKLEKRLA DKKAG-PIEF VSDN--PLFS FQAIIDPEDK
C_remanei_BCA2                  HWLRRHGFNS -IKKLEKRLA DKTAG-PIEF VSDN--PLFS FQAIIDPEDK
C_brennerii_BCA2                HWLRRHGFNS -IKKLEKRLA DKKAG-PIQF VSDN--PLFS FQAVIDPEDK
C_elegans_NP_001041015          HWLRRHGFNS -IRKLEKRLA DKNAG-PIEF VSDN--PLFS FSAVIDPEDK
P_pacificus_BCA2                HWLRRHGYNS -LRKLEKRLA DDKAG-PLEF VSSN--PLFS FSAIIDAEGK
A_caninum_FC551456+FC550353     HWLRRHGFAS -LKKLEERLA DKTAK-PIKF VSDN--PSFS FEAVIDPEDK
B_floridae_210100262            SWLTQHGNST -LTRFERYEM DRLQ--PITF QGIS--PKEL WDAYVDPQCH
B_floridae_210094934            SWLTQHGNST -LTRFERYEM DRLQ--PITF QGIS--PKEL WDAYVDPQCR
M_senile_FC835283               SWITTHGKSS -LTKLDQLIV NKAKPVKLVF NE-DN-DAER FEATIDEN--
N_vectensis_XP_001632619        PWLKKTGASS -LTRFEKVDM SQEGGVKLLF ED-AT-GGEP MEVTIDEGNK
A_pectinifera_DB424979+DB44052  QWCRTHGVEA SLNKIQDLSA RTPSEGVMTF DVGAT-PIEA YFKDFDSKDE
C_clemensi_225719368            AWLYKHGMDS -LNKLND-KL TSPES-PLTF MKDT---QHE FEANMDNK--
L_salmonis_225713548            AWLQRNGMVS -FKRFCEMKK MGKED-SLIF MKNT---KHE FEARIDSQ--
T_adhaerens_190581916           DWIYRFASQT YTKWEKTTLV DRSNSDQPLH LEFNE-NGLK FEANINQN--
P_lividus_139313180+139245724   NWLHAYGSPA -LEKYQKHME NPEEE--VRF EGGGRKGVKM AAVIEDPDSK
S_purpuratus_XP_001189115       NWLRAYGSPA -LEKYERHME NPAEE--VTY EGGGRKGAKL SAVIED-NGK
```

```
                                201
S_kowalevskii_187043763         FGIEDKLSQV NCLQQLQNIS SYGFLKDRLE SGRVRLHAMW ----------
X_bocki_117195962               FQMVDKLSQI NCLQQLQNIS SWGFLRSKLE NNEVRLHAMW FDIYTGDHYM
D_pseudoobscura_125776717       FAIEDKLSQI NTLQQMSNIA SYGFLKARLE SHDLHIHALW FDIYTGDIYY
D_willistoni_194166182          FAIEDKLSQI NTLQQMSNIA SYGFLKARLE SHDLHIHALW FDIYTGDIYY
D_melanogaster_24645213         FALEDKLSQI NTLQQMSNIA SYGFLKARLE SHDLHIHALW FDIYTGDIYY
D_erecta_190652591              FTLEDKLSQI NTLQQMSNIA SYGFLKARLE SHDLHIHALW FDIYTGDIYY
D_virilis_194152748             FAIEDKLSQI NTLQQMSNVA SYGFLKTRLE THNLHVHALW FDIYTGDIYY
D_mojavensis_193916036          FAVEDKLSQI NTLQQMSNIA SYGFLKTRLE SHNLHIHALW FDIYTGDIYY
D_grimshawi_193894023           FAIEDKLSQI NTLQQMSNVA SYGFLKSRLE SHNLHIHALW FDIYTGDIYY
D_ananassae_190626360           FAVEDKLSQI NTLQQMSNIA SYGFLKARLE SHDLHIHALW FDIYTGDIYY
A_aegypti_157110803+77891004    FALEDKLSQV NTLQQIENVA SYGFLKKRLE SHDLHIHALW FDIYTGDIYY
A_gambiae_57968460              FAIEDKLSQV NTLQQIENVA SYGFLKRRLE SHDLHIHALW FDIYTGDIYF
C_quinquefasciatus_170043321    FAIEDKLSQV NTLQQIENIA SYGFLKKRLE SHDLHIHALW FDIYTGDIYY
T_castaneum_91084165            FTIEDKLSQI NTLQQLQNIA SYGFLKKRLE KHQLHIHALW FDIYTGEIYY
N_vitripennis_156547528         FAIEDKLSQI NTLQQLQNVA SYGFLKRRLE KHNLHVHALW FDIYTGDIYY
A_mellifera_110764310           FGVEDKLSQI NTLQQLQNIA SYGFLKKRLE RHDLHIHALW FDIYTGDIYY
A_pisum_193713675               LSVTDKLSQV NTLQQIQNIA SYDFLKKRLE TYDLHIHALW FDIYTGDVHY
D_pulex_FE417346+FE409868       FSLTDKLSQL NTLQQLQHIA SYSFIQSAIN SGRVHLHALW FDIYTGDIYV
H_medicinalis_EY481200+EY50505  FKPVDKLSQV NTLQQLQNIA SHPFLKKKLE IGKVRLHALW IDVYTGDFHM
C_elegans_NP_741809.1           LAMEDKLSQI NVLQQLINIC SHEFLKEYLE SGRLHIHGMW FDIYKGEDYL
C_brennerii_BCA1                LPVEDKLSQI NVLQQLINIC SHEFLKEYLE SGRLHIHGMW FDIYKGEDYL
C_japonica_BCA1                 LPVEDKLSQI NVLQQLINIC SHEFLKEYLE SGRLHIHGMW FDIYKGEDYL
C_remanei_BCA1                  LSAEDKLSQI NVLQQLVNIC SHQILQEHLE SGRLHIHGMW FDVYTGDDYL
C_briggsae_A8XKV0               WSAEDKLSQI NVLQQIMNIS THEFLKDYLE AGNLHLHGAW FNIYDGEVFL
P_pacificus_BCA1                LKAEDKLSQI NVLQQLVNIA SHDALKEAFD QGKLHIHGMW FDVYKGEDYL
C_briggsae_BCA2                 LNVEDKLSQI NTLQQLENVA SHGFLKEFLE SQTVDLHAMW FDIYTGEMHM
C_remanei_BCA2                  LNVEDKLSQI NTLQQLENVA SHGFLKEFLE SQTVDLHAMW FDIYTGEMHM
C_brennerii_BCA2                LNVEDKLSQI NTLQQLENVA SHGFLKEFLE SQTVDLHAMW FDIYTGEMHM
C_elegans_NP_001041015          LNVEDKLSQI NTLQQLENVA SHGFLKEFLV SQTVDLHAMW FDIYTGEMHM
P_pacificus_BCA2                WNVEDRLSQI NTLQQLENIA SHGFLNEFLE SRQVDLHAMW FDVYAGEMYM
A_caninum_FC551456+FC550353     WGVEDKLSQI NTLQQLENCA SHGFLTEFLE KKTVDLHAMW FDIFAGEMYL
B_floridae_210100262            WTDQDQFSQV NVLQQLQNVS SHGFLKPRLK SGALQLHGMW LDSRQQLPYL
B_floridae_210094934            LTDQDQFSQV NVLQQLQNVS SHGFLKPRLK SGALQLHGMW LDSRQQLPYL
M_senile_FC835283               LNIQDKLSQV NVLQQLHNIG SYSFVREQLR AKQLNLLALW FDTANGEMYV
N_vectensis_XP_001632619        LDSVDKLSQV NVLQQLHNLK SFPFISNPLS KGALNLYGLW FDIKEGEMYM
A_pectinifera_DB424979+DB44052  KSLLDKLSKV NVLQQLEHLS SYRSIRRRVI AGTLSLHGMW YDVATDKIYW
C_clemensi_225719368            LLESDQLSQI NTLVQIENIY SYGFMKERMD QHQSVAHGLW LSLSSGEAHF
L_salmonis_225713548            LDEADQLSQI NTLVQMENIY SYDFMKNRID DKTAFVHGLW FSLTTGDVHY
T_adhaerens_190581916           LLPKDQLSQI NTLQQLLHVN SYSFMKEKIA AGTVKLYSLW FDIKDATCYV
P_lividus_139313180+139245724   LSNTDRLAQI NVLQQLEHLQ SYDFIGKRME TDQIRLHATF YDTVTGHMSV
S_purpuratus_XP_001189115       LSKTDRLAQI NVLQQLEHLQ SYDFIGKRME TDQIRLHATF YDTFSGNVYV
```

```
                                251
S_kowalevskii_187043763         ---------- ---------- ---------- ---------- ----
X_bocki_117195962               FSAKHGKRFV EISEENLEHL LVDSL----- ---------- ----
D_pseudoobscura_125776717       FSRGA-KQFI AVDEDSVDRL SAEVRRFYS- ---------- ----
D_willistoni_194166182          FSRGA-KCFV AVDEDSVERL SKEVRQFYS- ---------- ----
D_melanogaster_24645213         FSRGA-KRFL PVDEDTVDRL SEEVRRFYS- ---------- ----
D_erecta_190652591              FSRGA-KRFL PVDEDTVDRL SEEVRRFYS- ---------- ----
D_virilis_194152748             FSRGA-KRFI AVDESSVDQL SAEVRRFYS- ---------- ----
D_mojavensis_193916036          FSRGA-KRFV AVDESSVDQL SAEVRRFYS- ---------- ----
D_grimshawi_193894023           FSRGA-KRFI AVDESSVEQL SAEVRRFYS- ---------- ----
D_ananassae_190626360           FSRGA-KRFV AVDEKSVEEL SAEIRRFYS- ---------- ----
A_aegypti_157110803+77891004    FSRNS-KRFI PIDETSIEQL LDEVRRYYS- ---------- ----
A_gambiae_57968460              FSRNS-KRFI AIDESSIDRL LDEVRRYYS- ---------- ----
C_quinquefasciatus_170043321    FSRNS-KRFI PVDETTIERL LKEVNQFYS- ---------- ----
T_castaneum_91084165            FSRGA-KKFV VIDEENFPKL LQEVEKYYS- ---------- ----
N_vitripennis_156547528         FSRAN-KRFV EINELTETPL LKEIKKYYS- ---------- ----
A_mellifera_110764310           FSRAN-KKFV EINESTERCL LTEIKKYYS- ---------- ----
A_pisum_193713675               FSRQS-KQFV EINEKNVDGL VEEVSKYYC- ---------- ----
D_pulex_FE417346+FE409868       FSRKQ-KRFV EISEETSDYL LDEIREYFV- ---------- ----
H_medicinalis_EY481200+EY50505  FCRDS-NRFM LVNEESYENL LADGESNIDY ---------- ----
C_elegans_NP_741809.1           FSKDK-KRFV VIDEKTVTDL LAELNARYPV PEDQDGPVAF AKSN
C_brennerii_BCA1                FSKDK-KRFV VIDEKTVTDL LAELNTRYPL PEDQDGPVAF AQSN
C_japonica_BCA1                 S-QDK-K-IC VIDENTLTDL LAELNSRYPX PEDKDGAVAF AQSN
C_remanei_BCA1                  FSKDK-KRFV VIDEKTVDKH LSELNARCPL PEDQDGPVAF AKA-
C_briggsae_A8XKV0               FSKDR-KRFV VIDEKTVPSL SAELERRCPL PEDKAGDVVI QNLH
P_pacificus_BCA1                FSKEK-RQFV II-------- ---------- ---------- ----
C_briggsae_BCA2                 FSKPN-NRFV LIEESNVEEL IDEVEKHQ-- ---------- ----
C_remanei_BCA2                  FSKPN-NRFV LVDESNVEEL IDEVEKHQ-- ---------- ----
C_brennerii_BCA2                FSKPN-KRFV LVDESNVEEL IEEVEKHQ-- ---------- ----
C_elegans_NP_001041015          FSKPN-KQFV LVDESNVEEL IDEVEKHQT- ---------- ----
P_pacificus_BCA2                FSKPR-KQ-- ---------- ---------- ---------- ----
A_caninum_FC551456+FC550353     FSKPR-RKFI LIDEGTVDKL EEEVNQHKA- ---------- ----
B_floridae_210100262            FSKEQ-QRFV QITDNNIDSL L--------- ---------- ----
B_floridae_210094934            FSKEQ-QRFV QITDNNIDSL LK-------- ---------- ----
M_senile_FC835283               FSKQS-GTFE LITDNTLL-- ---------- ---------- ----
N_vectensis_XP_001632619        FSRKQ-KKFV LINKDTVNNL CSEVD----- ---------- ----
A_pectinifera_DB424979+DB44052  FSRAR-RMFL EINE------ ---------- ---------- ----
C_clemensi_225719368            FSKKD-KAFV NVTEDNVEEL VCR------- ---------- ----
L_salmonis_225713548            FLKNE-KVFI NVSEDNIDNL VQRSEE---- ---------- ----
T_adhaerens_190581916           FNKRD-KLFQ PIVSEEMLKS VSEFQINQK- ---------- ----
P_lividus_139313180+139245724   FSQSQ-GRFN FLPTEDFSSL SHYIFELKSS ---------- ----
S_purpuratus_XP_001189115       FNQKQ-GRFN FLPTADIDSL SHYIFQLRSS ---------- ----
```
